# Huoxue Qianyang Qutan Recipe limits cardiac remodeling by regulating FUNDC1/IP3R2 signaling pathway in obese hypertensive rats

**DOI:** 10.1038/s41598-026-56197-y

**Published:** 2026-06-04

**Authors:** Chunlei Hou, Yuxiu Zhao, Yulong Ma, Xunjie Zhou, Lei Yao, Jianhua Li, Mingtai Gui, Mingzhu Wang, Xiaozhe Chen, Deyu Fu, Bo Lu

**Affiliations:** Department of Cardiology, Yueyang Hospital of Integrated Traditional Chinese and Western Medicine, Shanghai University of Traditional Chinese Medicine, Shanghai, China

**Keywords:** Obesity, Hypertension, HuoXue QianYang QuTan Recipe, FUNDC1, Mitochondria-associated endoplasmic reticulum membranes, Cardiology, Cell biology, Physiology

## Abstract

Obese hypertension (OBH) increases cardiovascular risk through myocardial remodeling, which is associated with disrupted mitochondrial Ca²⁺ homeostasis and dysfunction of mitochondria-associated endoplasmic reticulum membranes (MAM). Huoxue Qianyang Qutan Recipe (HQQR) has been shown to lower blood pressure in OBH, but its mechanism related to MAM remains unclear. In this study, phenotypic assessments in OBH rats included blood pressure, morphological parameters, and cardiac ultrasound. Cardiomyocyte hypertrophy and mitochondrial Ca²⁺ levels were analyzed via pathological staining (assessed using fluorescence-based staining), while MAM ultrastructure was examined by electron microscopy. Additionally, the activity of mitochondrial respiratory chain Complexes I–IV was detected in vivo to evaluate mitochondrial respiratory function. In vitro, flow cytometry was used to evaluate mitochondrial Ca²⁺, mitochondrial reactive oxygen species (mitoROS), and membrane potential. Protein levels of FUNDC1 and IP3R2 were assessed. Co-immunoprecipitation revealed an interaction between FUNDC1 and IP3R2, and further experiments with FUNDC1 siRNA knockdown and overexpression were conducted to clarify the mechanism of HQQR. Results showed that HQQR significantly lowered blood pressure, reduced left ventricular mass, and alleviated cardiomyocyte hypertrophy in OBH rats. In vivo, HQQR altered MAM structure and function, facilitated mitochondrial Ca²⁺ transport, and modulated FUNDC1 and IP3R2 expression. In vitro, HQQR reduced mitoROS and preserved mitochondrial Ca²⁺ homeostasis. Both HQQR treatment and FUNDC1 knockdown attenuated angiotensin II-induced cardiomyocyte hypertrophy and mitochondrial damage, as indicated by decreased levels of ANP, BNP, β-MHC, mitochondrial Ca²⁺, and mitoROS. In contrast, FUNDC1 overexpression diminished the protective effects of HQQR. The interaction between FUNDC1 and IP3R2 was confirmed, and the decrease in IP3R2 may be associated with ubiquitination-mediated degradation. In conclusion, HQQR mitigates MAM dysfunction in OBH-induced myocardial remodeling by regulating the FUNDC1-IP3R2 interaction and promoting ubiquitin-dependent degradation of IP3R2, thereby maintaining mitochondrial Ca²⁺ homeostasis in cardiomyocytes.

**Supplementary Information:**

The online version contains supplementary material available at 10.1038/s41598-026-56197-y.

## Introduction

The global prevalence of obesity and hypertension has reached alarming levels, with projections indicating that over 4 billion people—exceeding half of the global population—will be overweight or obese by 2035^1^. This public health epidemic may incur economic losses exceeding $4 trillion due to reduced labor productivity. In China, obesity rates among women rose nearly fourfold from 2.0% in 1990 to 7.8% in 2022. Hypertension currently affects about 1.3 billion people worldwide^2^, and remains the leading cause to cardiovascular mortality. Notably, 65–78% of essential hypertension cases are attributed to obesity, which acts through multiple mechanisms including neurohormonal activation, inflammatory pathway activation, and renal dysfunction^3^. The co-occurrence of obesity and hypertension, termed obese hypertension (OBH), accelerates myocardial remodeling, substantially increasing cardiovascular risk^4,5^.

The pathophysiological mechanisms driving OBH-induced myocardial remodeling involve intricate and interconnected signaling cascades^6–8^. Emerging evidence underscores mitochondrial dysfunction and impaired energy metabolism as central contributors to OBH-related cardiac remodeling, particularly through mitochondria-associated endoplasmic reticulum membranes (MAM)-mediated Ca²⁺ signaling^9,10^. MAM forms specialized membrane contact sites that physically and functionally couple the endoplasmic reticulum (ER) with mitochondria. These dynamic junctions facilitate regulated Ca²⁺ transfer between organelles via protein-protein interactions^11,12^, thereby maintaining critical mitochondrial processes. Pathological MAM dysfunction disrupts Ca²⁺ homeostasis, leading to chronic mitochondrial Ca²⁺ overload. This dysregulation triggers a cascade of events including metabolic imbalance, oxidative stress resulting from mitochondrial reactive oxygen species (mitoROS) overproduction, mitochondrial structural damage, and subsequent mitochondrial DNA (mtDNA) leakage^13,14^. The inositol 1,4,5-trisphosphate receptor type 2 (IP3R2), a transmembrane protein located in the ER membrane, is essential for MAM formation. FUN14 domain-containing protein 1 (FUNDC1), a mitochondrial outer membrane protein enriched at MAM interfaces, has emerged as a critical modulator of cardiovascular function. In cardiomyocytes, FUNDC1 directly interacts with IP3R2 to regulate ER-derived Ca²⁺ flux into mitochondria and cytosol, thereby facilitating MAM assembly and Ca²⁺ signaling^15^. A previous study showed elevated expression of FUNDC1 and IP3R2 in diabetic heart tissue, where they mediate mitophagy in cardiomyocytes^16^. Additionally, FUNDC1-associated MAM contributes substantially to neovascularization processes^17^. Conversely, high-fat diet (HFD)-fed mice demonstrate reduced FUNDC1 expression, and genetic ablation of FUNDC1 exacerbates diet-induced cardiac hypertrophy, fibrosis, and dysfunction^18^. Despite these advances, the mechanistic links between MAM-mediated FUNDC1/IP3R2 regulation of mitochondrial Ca²⁺ dynamics in OBH-related heart disease—particularly in the context of traditional Chinese medicine (TCM) interventions—remain poorly understood. Pharmacological modulation of the FUNDC1/IP3R2 pathway could thus represent a viable strategy for attenuating myocardial remodeling.

In recent years, TCM has demonstrated promising effects in preventing and treating hypertension-related target organ damage^19,20^. The clinically established Huoxue Qianyang Qutan recipe (HQQR), with over 20 years of clinical application, effectively lowers blood pressure while improving glycolipid metabolism and reducing inflammation in hypertensive patients^21^. Emerging evidence demonstrates that HQQR confers significant organ protection, especially against cardiac fibrosis and cardiomyocyte hypertrophy in OBH. These effects may stem from the formulation’s modulation of ER stress and mitochondrial function^22–25^.

This study investigated how HQQR mitigates myocardial remodeling in OBH. By elucidating the molecular mechanisms underlying the therapeutic effects of HQQR, we aim to provide novel insights for the treatment of OBH-induced myocardial remodeling and related cardiovascular complications.

## Materials and methods

### Experimental animals and drug treatments

Five-week-old male spontaneously hypertensive rats (SHR, *n* = 78) and age-matched Wistar-Kyoto rats (WKY, *n* = 6) were purchased from Beijing Vital River Laboratory Animal Technology Co., Ltd. [license number: SCXK (Beijing) 2021-0006]. All animals were housed under controlled conditions (temperature: 22 ± 2 °C; relative humidity: 50–60%) at the experimental animal center of Yueyang Hospital of Integrated Traditional Chinese and Western Medicine. All animal procedures were approved by the Institutional Animal Care and Use Committee of Yueyang Hospital (approval number: YYLAC-2022-172) and conducted in accordance with the National Institutes of Health Guide for the Care and Use of Laboratory Animals. Of the 78 SHRs, 72 were fed a HFD (Jiangsu Medicine Biomedical Co., Ltd.) to induce obesity. The HFD consisted of 60% standard chow, 12.5% lard, 10% sucrose, 10% egg yolk powder, 5% milk powder, 2% cholesterol, and 0.5% bile salts. After 10 weeks, SHRs in the top 1/3 of body weight were identified as OBH rats based on previous reports^26,27^. The remaining 6 SHRs, along with all 6 WKY rats, were fed a standard normal diet (ND; supplied by Jiangsu Medicine Biomedical Co., Ltd.) throughout the study.

OBH rats were randomly divided into four groups by random number table (*n* = 6 per group): OBH group (SHRs fed HFD), OBH+HQQR-L group (OBH rats fed HFD and administered a low dose of HQQR at 19.35 g crude drug/kg/day via intragastric gavage), OBH+HQQR-H group (OBH rats fed HFD and administered a high dose of HQQR at 38.7 g crude drug/kg/day via intragastric gavage), and OBH+valsartan group (OBH rats fed HFD and administered valsartan at 30 mg/kg/day via intragastric gavage). WKY group (WKY rats fed ND) and SHR group (SHRs fed ND) served as controls. The sample size was determined based on our previous experiments, previously published studies in obesity hypertension or metabolic syndrome characterized by obesity and hypertension^24,28,29^.Valsartan capsules (batch number: X3230) were obtained from Beijing Novartis Pharmaceutical Co. Ltd. The dosages of HQQR and valsartan were determined based on our previous studies^23^. Valsartan, an angiotensin II (Ang II) receptor blocker, has been confirmed to exert cardiovascular protective effects in metabolic syndrome characterized by obesity and hypertension^30^. It can alleviate myocardial hypertrophy in hypertensive models by improving myocardial autophagy, inhibiting mitochondrial biosynthesis, and other mechanisms^31^. Therefore, valsartan was selected as the positive control drug in this study. Blinding was applied to both operators and data analysts during the experiment.

### Preparation of HQQR

HQQR was prepared by the Department of Pharmacy, Eastern Theater General Hospital, People’s Liberation Army, Nanjing, China. The formulation comprises seven herbal components: *Salvia miltiorrhiza* Bunge (common name: Dan Shen), *Cassiae* Semen (common name: Cassia seed), *Ligusticum chuanxiong* Hort (common name: Chuan Xiong), *Uncaria rhynchophylla* (Miq.) Miq. ex Havil. (common name: Gou Teng), *Taxillus chinensis* (DC.) Danser (common name: Sang Ji Sheng), *Crataegus pinnatifida* Bunge (common name: Shan Zha), and *Stigma* Maydis(common name: Yu Mi Xu), in a dry weight ratio of 5:10:3:5:5:5:10.

The extraction and powder preparation were performed as follows: First, Cassia Seed was decocted alone in water for 1 h. The remaining herbs (except *Uncaria rhynchophylla*) were then combined and decocted twice: initially with 10 volumes of water for 1 h, followed by a second decoction with 8 volumes of water for 40 min. The two decoctions were combined, allowed to precipitate, and filtered. *Uncaria rhynchophylla* was then added to the combined filtrate with 8 volumes of water, decocted for 40 min, and filtered. All filtrates were pooled and left to stand overnight, after which they were filtered again. The final filtrate was concentrated under reduced pressure, transferred to a vacuum drying oven, and dried at 80 °C. The dried extract was milled and passed through a 100-mesh sieve to obtain the HQQR fine powder. The formulation ratio and experimental doses were determined based on our previous studies^23^.

### Determination of components in HQQR and network pharmacology analysis

Methanol, acetonitrile, formic acid and isopropyl alcohol were purchased from ANPEL. All solvents were Liquid Chromatography-Mass Spectrometry (LC-MS/MS) grade. Ultra-pure water was prepared in-house using a Milli-Q water purification system (Millipore, Bedford, MA, USA). Components in HQQR and HQQR-containing serum were analyzed by UPLC-QE-Orbitrap-MS/MS. Detailed methods were provided in the Supplementary Materials. The detailed parameters of the mass spectrometer were listed in Table S1.

Potential targets of main compounds in HQQR were collected from TCM systematic pharmacology analysis platform (TCMSP, http://lsp.nwu.edu.cn/tcmsp.php)^32^, STITCH database (STITCH, https://stitch.embl.de/)^33^, PubChem chemistry database (PubChem, https://pubchem.ncbi.nlm.nih.gov/)^34^, and the universal protein resource (UniProt, https://www.uniprot.org/)^35^. Targets associated with OBH were collected by searching the human gene database (GeneCards, https://www.genecards.org/)^36^, DisGeNET database (DisGeNET, http://www.disgenet.org/)^37^, and Uniprot. The core targets of active ingredients of HQQR were screened using the Merge function, and the key active ingredients-core targets network was drawn to analyze the potential mechanisms of HQQR in the treatment of OBH. Detailed methods were in the Supplementary Materials.

### Assessment of physiological parameters and cardiac morphology

Systolic blood pressure (SBP), diastolic blood pressure (DBP), and mean blood pressure (MBP) were monitored bi-weekly using a non-invasive tail-cuff system (BP-98 A, Beijing Softron Biotechnology Co., Ltd., China). Prior to measurement, rats were acclimated at 35 °C for 5 min. Three consecutive measurements were taken, and the average value was calculated. Body weight was recorded bi-weekly, and body length was measured at the end of the treatment period. Lee’s index used to evaluate obesity in rats is similar to BMI (body mass index) in humans. Lee’s index was calculated as^38^: Lee ’s index= $\:\frac{{\sqrt[3]{\mathrm{b}\mathrm{o}\mathrm{d}\text{y }\:\mathrm{w}\mathrm{e}\mathrm{i}\mathrm{g}\mathrm{h}\mathrm{t}\:\left(\mathrm{g}\right)}\times\:10}^{3}}{\mathrm{n}\mathrm{o}\mathrm{s}\text{e }\:\mathrm{t}\text{o }\:\mathrm{a}\mathrm{n}\mathrm{u}\text{s }\:\mathrm{l}\mathrm{e}\mathrm{n}\mathrm{g}\mathrm{t}\mathrm{h}\:\left(\mathrm{c}\mathrm{m}\right)}.$

After 10 weeks of treatment, cardiac function was assessed by transthoracic echocardiography. Rats were anesthetized with 2% isoflurane to maintain a heart rate between 400 and 650 beats per minute. Cardiac parameters were measured using a Vevo 2100 high-resolution imaging system (Visual Sonics Inc., Canada). Measurements, including interventricular septal thickness at end-diastole (IVSD), left ventricular internal diameter at end-diastole (LVIDD), left ventricular posterior wall thickness at end-diastole (LVPWD), ejection fraction (EF) and fractional shortening (FS), were averaged across five consecutive cardiac cycles. Corrected left ventricular mass (LVM) was automatically calculated by the system’s built-in software.

Subsequently, rats were euthanized. Blood samples were collected from the abdominal aorta, and heart and liver tissues were rapidly harvested. The liver weight, left ventricular mass index (LVMI), heart weight-to-body weight ratio (HW/BW), liver weight-to-body weight ratio (LW/BW), and heart weight-to-tibia length ratio (HW/TL) were then calculated.

### Histological analysis

Left ventricular tissues were fixed, paraffin-embedded, and sectioned. Sections were stained with hematoxylin and eosin (H&E) and wheat germ agglutinin (WGA) following standard protocols. Images were captured using a light microscope (Nikon, Japan) at 200× magnification for morphological assessment.

### Ultrastructure and separation of MAM

For transmission electron microscopy (TEM), heart tissue was fixed in 2.5% glutaraldehyde, postfixed in 1% osmium tetroxide, and embedded in epoxy resin. Ultrathin sections were stained and observed under a TEM (Talos L120C, Thermo Fisher Scientific). MAM distance and length were quantified using ImageJ. MAM was isolated from heart tissues by differential ultracentrifugation as described^39^.

### Mitochondrial Ca^2+^ and membrane potential measurement

Mitochondrial Ca²⁺ levels were determined using Rhod-2 AM (Thermo, R1244). For histological analysis, rat cardiac tissue sections were fixed, permeabilized, and blocked, followed by incubation with Rhod-2 AM overnight at 4 °C. After washing, sections were incubated with an Alexa Fluor 488-conjugated secondary antibody (Beyotime, A0423) for 2 h at room temperature and imaged using a fluorescence microscope (Nikon, Japan) at 200× magnification. Five fields of view were randomly selected from each section. Background subtraction was performed before quantifying the mean fluorescence intensity with ImageJ.

For flow cytometric analysis, a 5 µM working solution of Rhod-2 AM was prepared in Hank’s Balanced Salt Solution (HBSS). Cells were harvested, washed with PBS, and incubated with the Rhod-2 AM working solution at 37 °C for 30 min. After washing, cells were further incubated in HBSS at 37 °C for 30 min to allow complete deesterification, and then resuspended in flow assay buffer. Mitochondrial Ca²⁺ levels were quantified using a flow cytometer.

Mitochondrial membrane potential was assessed using the JC-1 assay kit (Beyotime, C2006) according to the manufacturer’s instructions.

### Cell culture and treatment

Primary cardiomyocytes were isolated from 1-3-day-old Sprague-Dawley rats and maintained in Dulbecco’s Modified Eagle Medium supplemented with 10% fetal bovine serum in a humidified atmosphere of 5% CO_2_ and 95% air at 37 °C. All culture media were supplemented with penicillin (100 U/mL) and streptomycin (100 µg/mL). Cardiomyocytes were divided into the following five groups (Control, Ang II, Ang II + HQQR-L, Ang II + HQQR-H, and Ang II + Val). The control group was cultured in complete medium containing FBS.

Cardiac hypertrophy was induced with 1 µM Ang II (MCE, HY-13948) for 24 h. Cell Counting Kit-8 (Beyotime, C0039) was utilized to assess cell proliferation to determine the concentration of Ang II. Cells were seeded into 96-well plates at a density of 5 × 10^3^ cells per well. After being treated under different concentrations of Ang II (0 µM, 0.001 µM, 0.01 µM, 0.1 µM, 1 µM, 10 µM, and 100 µM) for 24 h, the cells were mixed with the CCK-8 solution and incubated in a 5% CO2 incubator for 1 h at 37 ℃. The optical density (OD) value was read at 450 nm using a microplate reader (DNM-9602, Prelong, Beijing, China).

For the three drug-intervention groups, Ang II-stimulated cardiomyocytes were co-treated with low-dose HQQR (0.2 mg/mL), high-dose HQQR (0.5 mg/mL) or valsartan (10 µM), respectively. The working concentrations of HQQR were determined according to our previous research^23^.

### Transfection of FUNDC1

FUNDC1 overexpression plasmids were purchased from General Biology Co. Ltd. (Chuzhou, China). FUNDC1 shRNAs and the corresponding negative control shRNA (shNC) were procured from GenePharma Co., Ltd. (Suzhou, China). Cardiomyocytes were transfected with shRNAs or overexpression plasmids via Lipofectamine 2000 (Thermo) following the manufacturer’s standard protocols. The sequences of shFUNDC1-1, shFUNDC1-2, and shFUNDC1-3 were listed in Table 1.

**Table 1:** Target sites and oligonucleotide sequences for FUNDC1.

Target gene	Target Site	Sequence
Fundc1	Fundc1-rat-167	GAGAGCGATGACGAGTCTTAC
	Fundc1-rat-281	GTAGCTACCCAGATAGTAATG
	Fundc1-rat-411	GCTATGTGCAGATCGACTGGA

### Western blot

Total protein concentration was measured with a BCA assay kit (Elabscience, E-BC-K318-M). Protein samples were resolved by SDS-PAGE and electrotransferred onto polyvinylidene difluoride (PVDF) membranes. The membranes were blocked with 5% skimmed milk at room temperature for 1 h and incubated overnight at 4 ℃ with primary antibodies: anti-FUNDC1 (CST, #49240), anti-IP3R2 (Santa, sc-398434), anti-atrial natriuretic peptide (ANP) (Abcam, ab225844), anti-brain natriuretic peptide (BNP) (Abcam, ab19645), anti-β-myosin heavy chain (β-MHC) (Abcam, ab37484), anti-GAPDH (Proteintech, 60009-1-AP), anti-Calreticulin (Abcam, ab92516), and anti-ubiquitin (Ub) (Proteintech, 10201-2-AP). Following three washes with TBST, the membranes were incubated with secondary antibodies at 37 °C for 1 h. Protein bands were visualized using a Tanon 5200 chemiluminescence imaging system (Shanghai, China) and quantified using ImageJ. The Western blot assay was performed in triplicate for each group.

### Co-immunoprecipitation (Co-IP)

Co-IP assays were carried out using the BersinBio Co-IP Kit (Cat. No. Bes3011, China) following the manufacturer’s protocols. Ang II-stimulated cardiomyocytes were lysed with ice-cold lysis buffer (Beyotime, P0038). Cell lysates were clarified by centrifugation. An aliquot of 300 µL supernatant was incubated overnight with primary antibodies against the target proteins at 4 °C under continuous rotation. Next, 100 µL Protein A/G-Plus-Agarose beads were added, and the mixture was incubated on a vertical rotator for 1–2 h at 4 °C. The immunoprecipitated complexes were harvested via centrifugation, then washed three times with wash buffer (10 min per wash) at 4 °C. Finally, the complexes were heated in 5× loading buffer to elute bound proteins. The eluted protein samples were subjected to Western blot analysis as detailed in Sect.  2.10.

### MitoROS measurement

Mitochondrial superoxide production in frozen rat cardiac tissue sections was detected with MitoSOX™ Red mitochondrial superoxide indicator (Thermo Fisher Scientific, M36008, Waltham, MA, USA) following the manufacturer’s protocols. Rat hearts were quickly excised, rinsed with ice-cold PBS, embedded in OCT compound (Tissue-Tek, Sakura, Japan), and flash-frozen in liquid nitrogen.

The frozen tissues were sliced into 10–µm-thick sections using a cryostat. Sections were thaw-mounted on Superfrost Plus glass slides and stored at − 80 °C prior to staining. Fluorescence images were acquired with a fluorescence microscope (Nikon, Japan) at 200× magnification under consistent exposure parameters. MitoSOX™ Red fluorescence intensity from random fields was quantified via ImageJ software after background subtraction, and results were presented as relative fluorescence intensity.

For cellular samples, a stock solution of MitoSOX (10 µg/13 µL in DMSO) was diluted with serum-free DMEM to prepare the working solution. Cells were rinsed with PBS, digested with trypsin, and centrifuged at 1000 rpm for 5 min to harvest cell pellets. Cell pellets were resuspended in 1 mL of working solution and incubated at 37 °C in a 5% CO_2_ incubator for 30 min. After rinsing three times with serum-free DMEM, cells were analyzed by flow cytometry.

### Measurement of ATP content and mitochondrial respiratory chain complex I-IV

Intracellular ATP levels were determined with an ATP assay kit (Jiancheng Bioengineering Institute, A095, Nanjing, China). Briefly, cells were harvested and lysed by sonication. The cell suspension was centrifuged at 4000 rpm for 5 min, and the resulting supernatant was collected. After adding chromogenic reagent, the mixture was incubated at room temperature for 5 min. The absorbance was measured at 636 nm, and the ATP concentration was calculated based on the standard curve.

The enzymatic activities of mitochondrial respiratory chain complexes I to IV in myocardial tissues were determined via colorimetric assays. According to the manufacturer’s protocols, myocardial tissues were homogenized to prepare a 10% tissue homogenate before sample loading (Yuanye Biotech, Shanghai, China).

### Statistical analysis

All statistical analyses were performed using GraphPad Prism 10 (GraphPad Software, Boston, Massachusetts USA, www.graphpad.com). All data are expressed as the mean ± standard deviation (SD). Differences among multiple groups were evaluated by one-way analysis of variance (one-way ANOVA), and Fisher’s Least Significant Difference (LSD) test was used for post-hoc multiple comparisons.

## Results

### Identification of compounds in HQQR and network pharmacology study

LC-MS/MS fragmentation spectra were acquired to characterize chemical constituents of HQQR. In total, 1144 compounds were identified and summarized in Table S1. The base peak chromatograms of HQQR in positive and negative ion modes are displayed in Figure S1. The major constituents of HQQR are listed as follows: Hirsutine, Rhynchophylline, Dihydrotanshinone I, Tanshinone IIA, Senkyunolide A, Betaine, Chlorogenic acid, Isoquercitrin, Luteolin, Ferulic acid, Danshensu, Salvianolic acid A, Quercetin-3-gentiobioside, Cryptochlorogenic acid, Protocatechuic acid, Lithospermic acid, Salvianolic acid C, P-Coumaric acid, Gallic acid and Rosmarinic acid. Ion chromatograms of 20 representative compounds are presented in Figure S2–S3.

All 1144 compounds were classified into multiple major superclasses according to ClassyFire chemical taxonomy. The relative proportion of each superclass is illustrated in the pie chart based on ClassyFire ontology (Figure S4A). Phenylpropanoids and polyketides were the dominant groups (25%), followed by lipids and lipid-like molecules (23%) and organoheterocyclic compounds (14%).

A total of 426 candidate targets corresponding to the major compounds were retrieved. Furthermore, 7870 OBH-related targets were collected from GeneCards, UniProt and DisGeNET databases. After intersection analysis, 287 overlapping targets were screened out via the merge function in Cytoscape, which were regarded as the potential therapeutic targets of HQQR against OBH. A Venn diagram was constructed using jvenn^40^ to visualize compound targets, disease targets and their overlapping targets (Figure S4B).

The 287 intersecting targets were imported into the STRING database to generate a protein-protein interaction (PPI) network. Subsequent centrality analysis using CentiScape 2.2 identified 16 hub genes, including FUNDC1 (Figure S4C). Meanwhile, an active ingredient–target network was also established (Figure S4D).

KEGG pathway enrichment analysis showed that these core targets were markedly enriched in glycolysis and gluconeogenesis (Figure S4E). GO functional enrichment revealed significant changes in carboxylic acid metabolic process, circulatory system process, mitochondrial membrane component and Ca²⁺ channel activity (Fig. 4SF-G). Taken together, the network pharmacology findings strongly support our follow-up experiments focusing on Ca²⁺ signaling and mitochondrial dysfunction.

### HQQR reduced BP, Lee’s index, LW/BW, and alleviated LVH in OBH rats

**Fig. 1:**
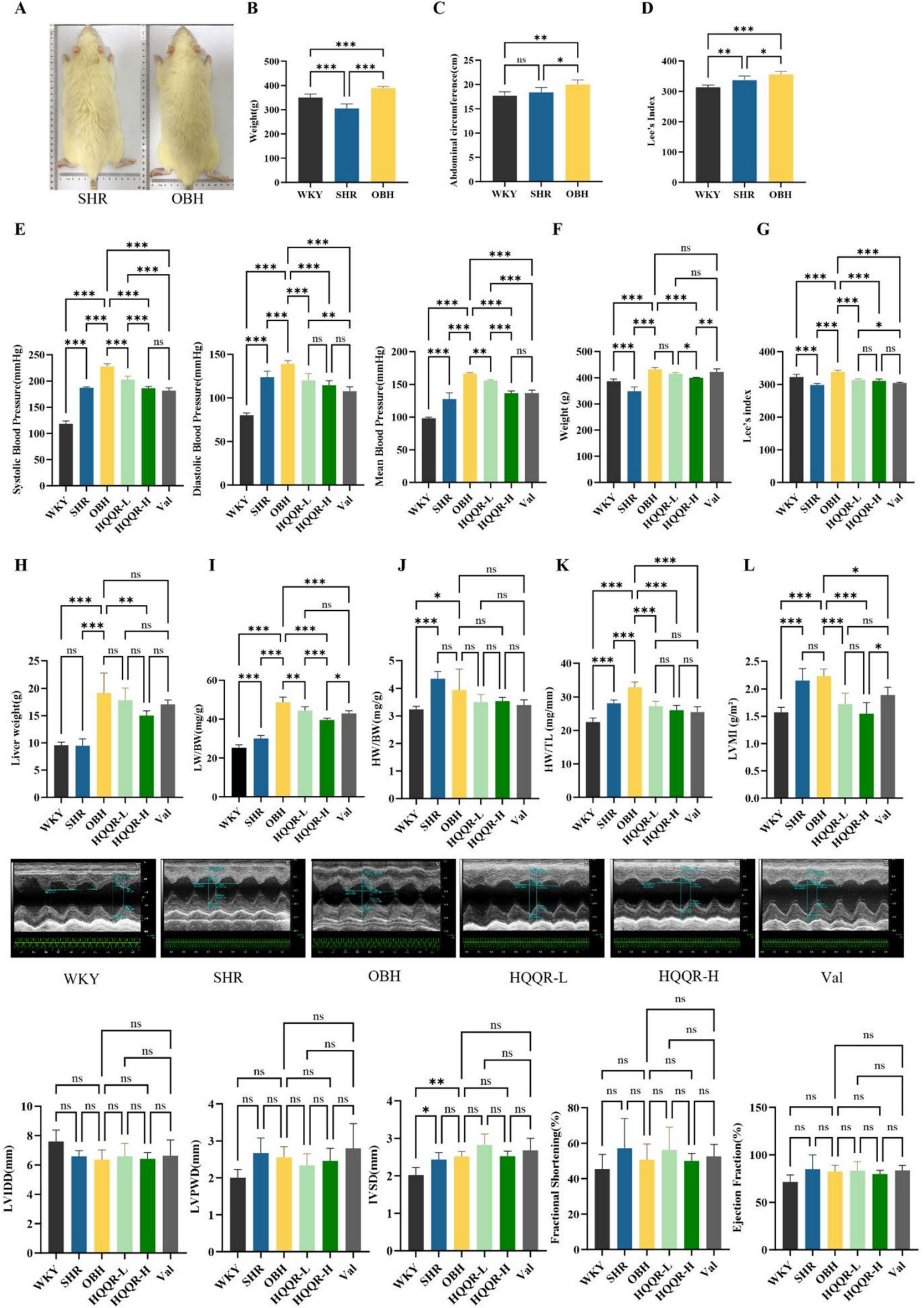
HQQR improves metabolic and cardiovascular parameters in OBH rats. (**A**) Representative images showing body size of SHR and OBH rats. (**B**–**D**) Body weight, abdominal circumference, and Lee’s index in different groups (*n* = 6). (**E**) SBP, DBP, and MAP after treatment (*n* = 6). (**F**,**G**) Body weight and Lee’s index after treatment (*n* = 6). (**H**,**I**) Liver weight and LW/BW after treatment (*n* = 6). (**J**,**K**) HW/BW and HW/TL after treatment (*n* = 6). (**L**) Representative echocardiographic images and quantitative analysis of cardiac function parameters, including LVMI, LVIDD, LVPWD, IVSD, EF, and FS (*n* = 6). Data are presented as mean ± SD. *, **, *** respectively means *P* < 0.05, *P* < 0.01, and *P* < 0.001. *WKY* Wistar–Kyoto rats, *SHR* spontaneously hypertensive rats, *OBH* obese hypertensive rats, *HQQR-L* low-dose Huoxue Qianyang Qutan Recipe, *HQQR-H* high-dose Huoxue Qianyang Qutan Recipe, *Val* valsartan, *LW/BW* liver weight/body weight, *HW/BW* heart weight/body weight, *HW/TL* heart weight/tibia length, *LVMI* left ventricular mass index, *LVIDD* left ventricular internal diameter at end-diastole, *LVPWD* left ventricular posterior wall thickness in diastole, *IVSD* interventricular septal thickness, *EF* ejection fraction, *FS* fractional shortening, *SBP* systolic blood pressure, *DBP* diastolic blood pressure, *MAP* mean arterial pressure.

OBH rats exhibited greater body size than SHR rats (Fig. 1A), as well as higher body weight (Fig. 1B), abdominal circumference (Fig. 1C), and Lee’s index (Fig. 1D), confirming the successful establishment of the OBH rat model induced by HFD. HQQR-L, HQQR-H, and valsartan all reduced elevated SBP, DBP, and MBP in OBH rats. The antihypertensive effect of HQQR was dose-dependent, with HQQR-H demonstrating a significantly better antihypertensive effect than HQQR-L. There was no significant difference in the antihypertensive effect between HQQR-H and valsartan (Fig. 1E). Furthermore, compared with the OBH group, HQQR-H reduced body weight (Fig. 1F), Lee’s index (Fig. 1G), liver weight (Fig. 1H) and LW/BW (Fig. 1I) significantly, while valsartan produced no significant changes in body weight or liver weight. High-dose HQQR could further reduce LW/BW compared with valsartan. HQQR-L, HQQR-H, and valsartan alleviated the HFD-induced increase in HW/BW and HW/TL in the OBH group (Fig. 1J-K), indicating that both HQQR (regardless of dose) and valsartan could effectively alleviate HFD-induced myocardial hypertrophy in OBH rats. Representative echocardiography results are shown in Fig. 1L. LVMI in the HQQR-H group was lower than that in the valsartan group, suggesting that high-dose HQQR had a superior effect in alleviating left ventricular hypertrophy compared to valsartan. Neither HQQR (HQQR-L or HQQR-H) nor valsartan had a significant effect on IVSD, LVIDD, LVPWD, EF, and FS. This suggests that cardiac systolic function remained intact in the early stage of obesity-associated hypertension, despite the presence of myocardial hypertrophy and metabolic abnormalities.

### HQQR altered MAM function in OBH rats

**Fig. 2:**
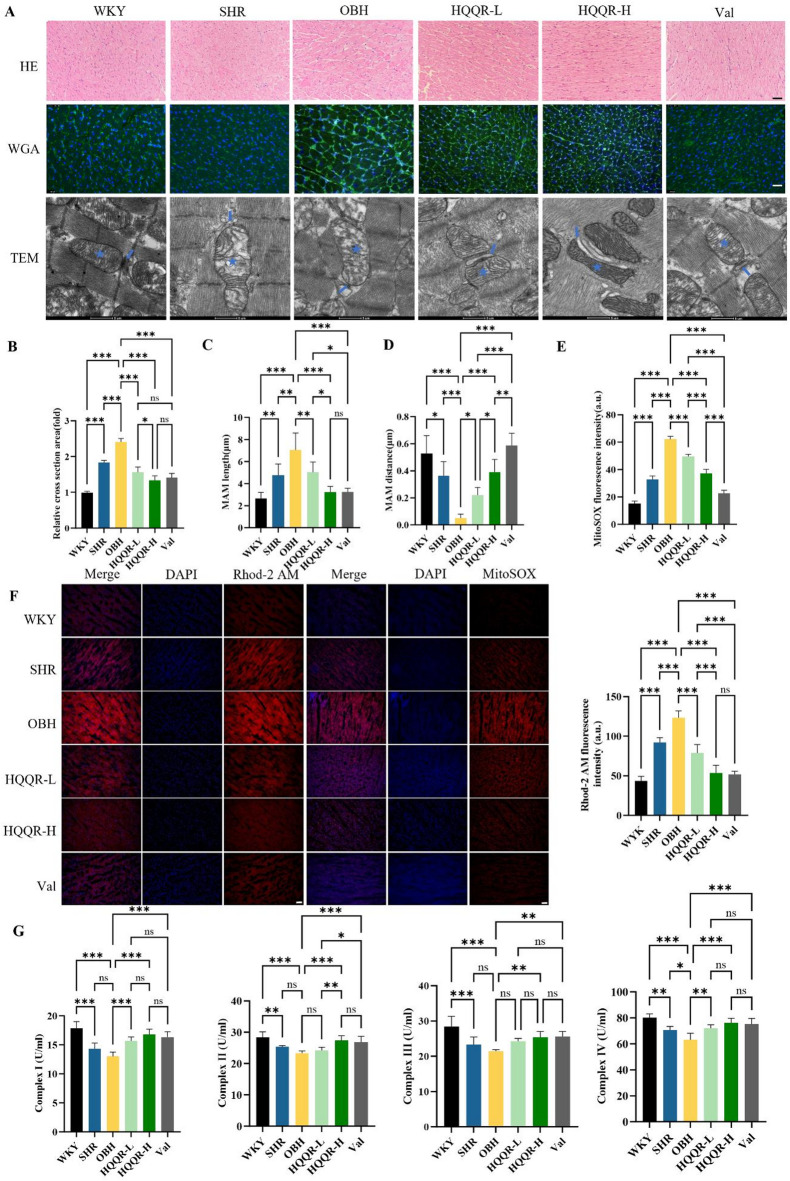
HQQR alleviated left ventricular hypertrophy and improved MAM function in OBH rats. (**A**) Representative images of HE, WGA, and TEM of myocardial tissue (*n* = 6; scale bars = 100 μm for HE and WGA; 5 μm for TEM). (**B**) Quantification of cardiomyocyte cross-sectional area (*n* = 6). (**C**,**D**) Quantitative analysis of MAM structure, including mitochondrial length and ER–mitochondria distance (*n* = 6). (**E**) Quantification of mitochondrial Ca²⁺ levels and mitoROS (*n* = 6). (**F**) Representative fluorescence images of mitochondrial Ca²⁺ and mitoROS (*n* = 6; scale bar = 100 μm). (**G**) Activities of mitochondrial electron transport chain complexes I–IV (*n* = 6 per group). Data are presented as mean ± SD. *, **, and *** respectively means *P* < 0.05, *P* < 0.01, and *P* < 0.001. WKY, Wistar–Kyoto rats; SHR, spontaneously hypertensive rats; OBH, obese hypertensive rats; HQQR-L, low-dose Huoxue Qianyang Qutan Recipe; HQQR-H, high-dose Huoxue Qianyang Qutan Recipe; Val, valsartan; HE, hematoxylin and eosin; WGA, wheat germ agglutinin; TEM, transmission electron microscopy; MAM, mitochondria-associated endoplasmic reticulum membrane; DAPI, 4′,6-diamidino-2-phenylindole; mitoROS, mitochondrial reactive oxygen species.

Histopathological examination revealed distinct myocardial injury patterns across groups. As shown in Fig. 2A, HE staining demonstrated mild myocardial fiber disarray and cardiomyocyte edema in SHRs. In contrast, OBH rats exhibited more severe edema, degenerative changes, and disorganization of myocardial architecture, alongside prominent inflammatory cell infiltration—key features of pathological myocardial inflammation that exacerbate cardiac damage in the obese hypertensive state. HQQR intervention reversed these pathological abnormalities in a dose‑dependent manner. The HQQR‑H group achieved significant improvement, with pathological manifestations approaching those of the valsartan‑treated group (Fig. 2A). Consistently, quantitative analysis of cardiomyocyte cross‑sectional area confirmed that HQQR treatment markedly reduced cardiac hypertrophy in OBH rats, with the HQQR‑H group exhibiting the most robust effect (Fig. 2B).

To further explore the underlying mechanisms, we focused on mitochondrial function and MAM structure. TEM analysis revealed striking ultrastructural abnormalities in OBH rats: the ER was swollen and aggregated, mitochondria were elongated and highly interconnected, and the ER–mitochondria distance was significantly reduced, indicative of excessive MAM formation (Fig. 2A, C–D). Such structural perturbations promoted aberrant ER–mitochondrial crosstalk, thereby accelerating myocardial injury. HQQR intervention, particularly at the high dose, restored normal mitochondrial morphology, reversed ER swelling, and increased the ER–mitochondria distance to a level similar to that observed with valsartan. Consistent with these structural improvements was that HQQR treatment restored mitochondrial function and stability. Figure 2E and F indicated that compared with the WKY control group, the mitochondrial Ca^2+^ levels and mitoROS production in OBH rats were significantly increased. HQQR treatment significantly alleviated these abnormal increases, and the Ca^2+^ levels in the HQQR-H group were comparable to those in the valsartan group. These findings suggest that HQQR can effectively alleviate mitochondrial Ca^2+^ overload and oxidative stress in OBH rats, representing an important cardiac protection mechanism. At the same time, HQQR treatment dose-dependently restored the activities of mitochondrial respiratory chain complexes I - IV, which were significantly impaired in OBH rats (Fig. 2G). In summary, these results indicated that HQQR improved mitochondrial oxidative phosphorylation function in a dose-dependent manner and restores the MAM structure to normal, thereby alleviating myocardial injury mediated by MAM in OBH rats.

### HQQR reduced the expression of FUNDC1 and IP3R2 while enhancing the ubiquitination of IP3R2

**Fig. 3:**
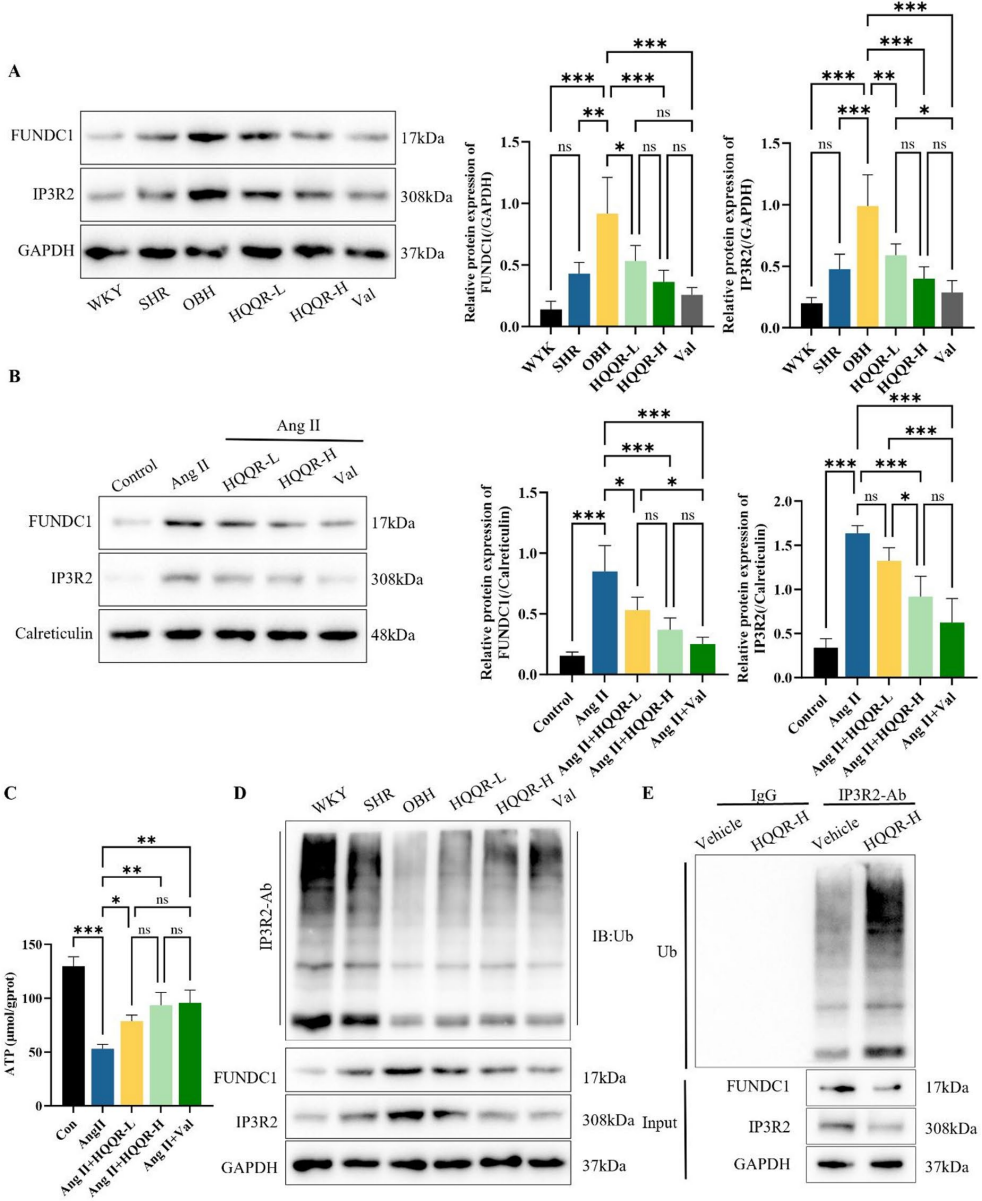
HQQR downregulated the expression of FUNDC1 and IP3R2 while promoting the ubiquitination of IP3R2. (**A**) Representative blot images and quantitative analysis of FUNDC1 and IP3R2 protein expression in rat myocardial tissue (*n* = 4). Original blots are presented in Original Blot Fig. 1. (**B**) Representative blot images and quantitative analysis of FUNDC1 and IP3R2 in primary cardiomyocytes following Ang II stimulation (*n* = 4). Original blots are presented in Original Blot Fig. 2. (**C**) ATP content in cardiomyocytes (*n* = 4). (**D**,**E**) Effect of HQQR treatment on IP3R2 ubiquitination levels in vivo and in vitro (*n* = 4). GAPDH was used as the loading control. Original blots are presented in Original Blot Fig. 3.Data are presented as mean ± SD. *, **, and *** respectively means *P* < 0.05, *P* < 0.01, and *P* < 0.001. *WKY* Wistar–Kyoto rats; *SHR* spontaneously hypertensive rats; *OBH* obese hypertensive rats; *HQQR-L* low-dose Huoxue Qianyang Qutan Recipe; *HQQR-H* high-dose Huoxue Qianyang Qutan Recipe; *Val* valsartan; *FUNDC1* FUN14 domain-containing protein 1; *IP3R2* inositol 1,4,5-trisphosphate receptor type 2; *GAPDH* glyceraldehyde-3-phosphate dehydrogenase; *Ang II* angiotensin II; *IB* immunoblotting; *Ub* ubiquitination.

Consistent with its protective effects on myocardial pathology and mitochondrial function, HQQR treatment reduced the elevated expression of FUNDC1 and IP3R2 in OBH rats (Fig. 3A). The effects of different concentrations of Ang II on cardiomyocytes were evaluated using the CCK-8 assay, and 1 µM Ang II was selected for subsequent experiments (Figure S5). This concentration was consistent with our previous research. In vitro, HQQR decreased the expression of FUNDC1 in Ang II-injured cardiomyocytes, with the high-dose group showing superior effects over the low-dose group. HQQR also reduced the expression of IP3R2, with the high-dose group demonstrating better efficacy than the low-dose group (Fig. 3B). Notably, FUNDC1 was identified as a core target of HQQR intervention in OBH via LC-MS/MS integrated network pharmacology analysis, which is in accordance with our in vivo and in vitro experimental results (Figure S4C). In line with these changes, we measured cellular ATP levels to assess the functional consequences of FUNDC1/IP3R2 modulation. As shown in Fig. 3C, Ang II stimulation significantly reduced ATP content in cardiomyocytes, reflecting impaired mitochondrial bioenergetics. HQQR treatment dose-dependently restored ATP production, with HQQR-H showing a more pronounced improvement than HQQR-L. The protective effect of HQQR-H was comparable to that of valsartan. Furthermore, the ubiquitination levels of IP3R2 in the OBH group were lower than those in the SHR and WKY groups, whereas HQQR and valsartan treatment improved the ubiquitination modification of IP3R2 (Fig. 3D). A similar phenomenon was observed in vitro, where HQQR-H increased the ubiquitination level of IP3R2 (Fig. 3E). These results indicate that HQQR may regulate IP3R2 expression by promoting its ubiquitination-dependent degradation, and jointly regulate FUNDC1 and IP3R2 to alleviate myocardial injury in OBH rats, which may be closely related to its regulation of mitochondrial and ER function observed in Fig. 2.

### Kockdown of FUNDC1 improved the effects of Ang II on cardiomyocytes

**Fig. 4:**
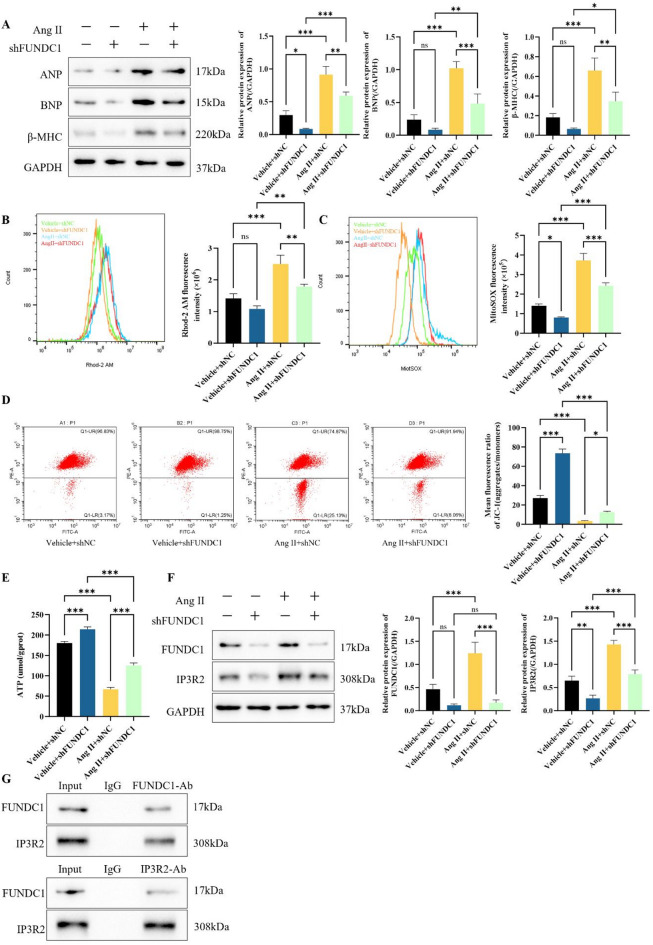
Kockdown of FUNDC1 improved the effects of Ang II on cardiomyocytes. (**A**) Representative immunoblot images with quantitative analysis of hypertrophy-related proteins, including ANP, BNP, and β-MHC (*n* = 3). Original blots are presented in Original Blot Fig. 4. (**B**) Mitochondrial Ca²⁺ levels (*n* = 3). (**C**) Quantification of mitoROS levels by flow cytometry (*n* = 3). (**D**,**E**) Mitochondrial membrane potential and ATP levels in primary cardiomyocytes (*n* = 3). (**F**) Representative immunoblot images and quantitative analysis of FUNDC1 and IP3R2 protein expression (*n* = 3). Original blots are presented in Original Blot Fig. 5. (**G**) Interaction between FUNDC1 and IP3R2 in cardiomyocytes detected by co-immunoprecipitation (*n* = 3). Original blots are presented in Original Blot Fig. 6. Data are presented as mean ± SD. *, **, and *** respectively means *P* < 0.05, *P* < 0.01, and *P* < 0.001. *Ang II* angiotensin II; *ANP* atrial natriuretic peptide; *BNP* brain natriuretic peptide; *β-MHC* β-myosin heavy chain; *GAPDH* glyceraldehyde-3-phosphate dehydrogenase; *FUNDC1* FUN14 domain-containing protein 1; *IP3R2* inositol 1,4,5-trisphosphate receptor type 2; *mitoROS* mitochondrial reactive oxygen species; *NC* negative control; *ATP* adenosine triphosphate.

WB analysis confirmed the successful FUNDC1 knockdown (Figure S6A). Consistent with the in vivo findings, indicators associated with cardiomyocyte hypertrophy, including ANP, BNP, and β-MHC, were pathologically elevated after treatment with Ang II, and these indicators decreased after HQQR treatment (Fig. 4A). To examine the effects of Ang II and FUNDC1 knockdown on mitochondrial function, levels of mitochondrial Ca²⁺ (Fig. 4B), mitoROS (Fig. 4C), and mitochondrial membrane potential (Fig. 4D) were measured. Mitochondrial Ca²⁺ concentration was significantly increased, accompanied by a decrease in JC-1 aggregate/monomer ratio, and mitochondrial membrane potential depolarization, indicating impaired mitochondrial Ca²⁺ handling and severe mitochondrial dysfunction. This imbalance in Ca²⁺ homeostasis was associated with increased mitochondrial oxidative stress, accompanied by a decrease in mitochondrial ATP levels (Fig. 4E), suggesting reduced energy metabolism function in damaged mitochondria. All these Ang II-induced abnormalities were reversed by FUNDC1 knockdown (Fig. 4A-E). Additionally, IP3R2 expression decreased concurrently with FUNDC1 inhibition (Fig. 4F), further supporting the coordinated regulation of FUNDC1 and IP3R2 observed in Fig. 3. Co-IP data demonstrated that FUNDC1 might directly bind to IP3R2 to tether the ER and mitochondria in Ang II-injured cardiomyocytes (Fig. 4G), which may explain how FUNDC1 knockdown modulates ER-mitochondrial Ca²⁺ transport and mitochondrial function, thereby alleviating Ang II-induced cardiomyocyte hypertrophy and mitochondrial dysfunction.

### HQQR alleviated cardiomyocyte injury via FUNDC1/IP3R2 signaling in primary cardiomyocytes

**Fig. 5:**
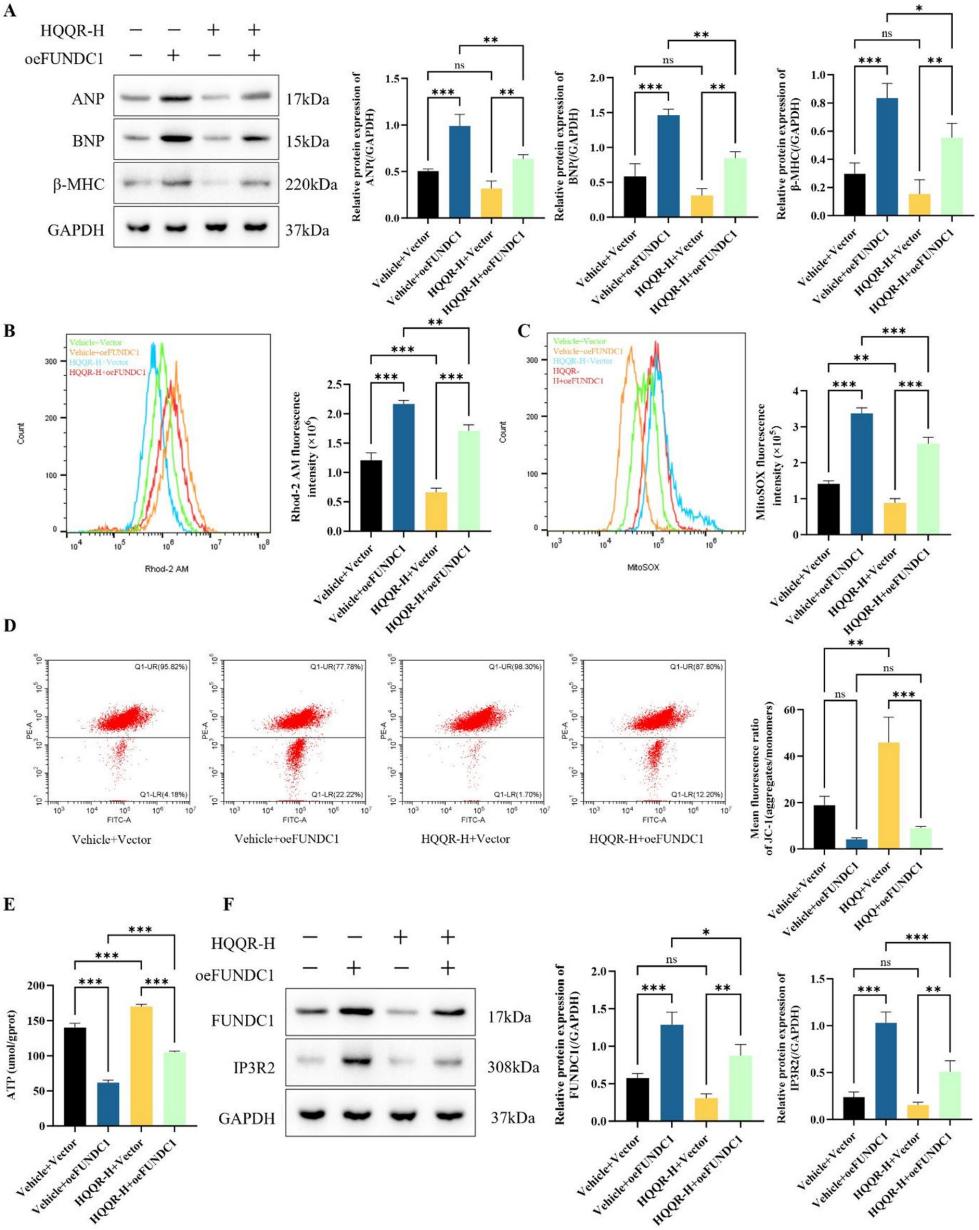
HQQR downregulates FUNDC1/IP3R2 expression and improves MAM function in primary cardiomyocytes. (**A**) Representative immunoblot images and quantitative analysis of hypertrophy-related proteins, including ANP, BNP, and β-MHC (*n* = 3). Original blots are presented in Original Blot Fig. 7. (**B**) Mitochondrial Ca²⁺ levels after treatment (*n* = 3). (**C**) Quantification of mitoROS after treatment by flow cytometry (*n* = 3). (**D**) Mitochondrial membrane potential level after treatment (*n* = 3). (**E**) ATP levels in primary cardiomyocytes (*n* = 3). (**F**) Representative immunoblot images and quantitative analysis of FUNDC1 and IP3R2 protein expression (*n* = 3). Original blots are presented in Original Blot Fig. 8. Data are presented as mean ± SD. *, **, and *** respectively means *P* < 0.05, *P* < 0.01, and *P* < 0.001. *HQQR-H* high-dose Huoxue Qianyang Qutan Recipe; *FUNDC1* FUN14 domain-containing protein 1; *IP3R2* inositol 1,4,5-trisphosphate receptor type 2; *GAPDH* glyceraldehyde-3-phosphate dehydrogenase; *ANP* atrial natriuretic peptide; *BNP* brain natriuretic peptide; *β-MHC* β-myosin heavy chain; *mitoROS* mitochondrial reactive oxygen species; *ATP* adenosine triphosphate.

To further confirm the regulatory role of FUNDC1 in HQQR-mediated myocardial protection, WB analysis erified efficient overexpression of FUNDC1 protein (Figure S6B). Consistent with the aforementioned in vitro findings, in Ang II-injured cardiomyocytes with FUNDC1 overexpression, HQQR significantly reduced the increased expression of ANP, BNP, and β-MHC (Fig. 5A), indicating that HQQR could alleviate cardiomyocyte hypertrophy under conditions of FUNDC1 overexpression. Furthermore, HQQR improved mitochondrial function by reducing mitochondrial Ca²⁺ levels (Fig. 5B), mitoROS production (Fig. 5C), mitochondrial membrane potential (Fig. 5D), and increasing ATP levels (Fig. 5E)—abnormalities induced by Ang II and FUNDC1 overexpression. Additionally, HQQR reduced the expression of IP3R2 in Ang II-injured cardiomyocytes with FUNDC1 overexpression (Fig. 5F). These results further demonstrated that HQQR exerts its myocardial protective effects by downregulating IP3R2 and improving mitochondrial function, even when FUNDC1 was overexpressed, suggesting that FUNDC1/IP3R2 served as key targets of HQQR in regulating myocardial hypertrophy and mitochondrial dysfunction.

## Discussion

Our study demonstrated that HQQR markedly ameliorates the core pathological manifestations of OBH in rats, including hypertension, visceral fat accumulation, and excessive weight gain. Notably, HQQR exhibits superior efficacy to valsartan, especially in improving the LW/BW ratio. Furthermore, HQQR alleviates myocardial edema, cellular degeneration, and myocardial fiber disorganization in OBH rats and blocks the development of cardiac hypertrophy. Mechanistically, HQQR preserves mitochondrial Ca²⁺ homeostasis in cardiomyocytes and downregulates the expression of FUNDC1 and IP3R2 in Ang II-injured cardiomyocytes, with high-dose HQQR exerting more potent effects. These results highlight the critical regulatory effect of HQQR on mitochondrial function and Ca²⁺ homeostasis. Collectively, HQQR improves systemic metabolic and hypertensive abnormalities in OBH rats and provides robust protective effects against adverse myocardial remodeling.

Mitochondria occupy roughly 30% of adult cardiomyocyte volumes and are essential for normal cardiac function^41^. Accumulating evidence implicates mitochondrial damage and impaired energy metabolism as central mechanisms in OBH-induced myocardial remodeling, processes intimately connected to intracellular Ca²⁺ signaling^42–44^. As a key second messenger, Ca²⁺ regulates diverse cellular activities including metabolism, differentiation, proliferation, and apoptosis^45,46^. In cardiomyocytes, dysregulation of Ca²⁺ homeostasis is closely associated with pathological states, such as myocardial hypertrophy and heart failure^47,48^. MAM serves as critical junctions between the ER and mitochondria, coordinating Ca²⁺ signaling, lipid metabolism, and mitochondrial dynamics^49–51^. Excessive communication between the ER and mitochondria can lead to rapid Ca²⁺ influx from the ER to the mitochondria via MAM, disrupting mitochondrial Ca²⁺ homeostasis, stimulating mitoROS production, and triggering mtDNA release^52–54^. These findings underscore the pivotal role of MAM in preserving the metabolic and physiological stability of cardiomyocytes^55^.

FUNDC1, a mitochondrial outer membrane protein localized in MAM, plays a critical role in MAM formation and maintenance^22^. Its deficiency reduces MAM protein abundance and disrupts mitochondrial-ER contacts, exacerbating mitochondrial dysfunction. The ER-resident IP3R2, a primary Ca²⁺ release channel, mediates rapid Ca²⁺ transfer between the ER and mitochondria while maintaining intracellular Ca²⁺ homeostasis, thereby facilitating MAM function^56^. Studies have shown that FUNDC1 and IP3R2 can form a protein complex that modulates MAM structure and function, influencing both Ca²⁺ homeostasis and mitochondrial function^15^. In OBH rats, we observed upregulation of FUNDC1 and IP3R2, key MAM regulators of Ca²⁺ homeostasis, suggesting that altered MAM structure and function promoted excessive communication between the ER and mitochondria and disrupts mitochondrial Ca²⁺ homeostasis during OBH-induced myocardial hypertrophy. Furthermore, HQQR treatment restored mitochondrial Ca²⁺ homeostasis while suppressing FUNDC1 and IP3R2 overexpression, thus maintaining the structure and function of MAM. The amelioration of MAM-mediated mitochondrial dysfunction may be an important mechanism for HQQR to improve myocardial remodeling in OBH. Mechanistically, HQQR modulated cardiomyocyte function through FUNDC1 downregulation, which enhanced IP3R2 ubiquitination and degradation, limiting pathological ER-to-mitochondrial Ca²⁺ transfer and preserving mitochondrial integrity. Genetic manipulation experiments demonstrated that FUNDC1 knockdown rescued Ang II-induced mitochondrial dysfunction in cardiomyocytes, whereas FUNDC1 overexpression worsened these mitochondrial defects, establishing the central role of FUNDC1 in HQQR mechanism of action and its regulatory control over IP3R2. Previous study showed that the binding of FUNDC1 to IP3R2 inhibited the interaction between IP3R2 and the E3 ubiquitin ligase RNF170, thereby suppressing the ubiquitination and proteasome-mediated degradation of IP3R2^57^. However, the regulatory role of RNF170 in this study remains to be further verified. In rat AR42J cells, six ubiquitination sites of IP3R2 including Lys-909, Lys-1589, Lys-1722, Lys-1836, Lys-1864 and Lys-2071 were characterized. Mechanistically, all IP3R isoforms exhibit conserved ubiquitination patterns across different cell types, which are modified by monoubiquitin, Lys-48- and Lys-63-linked ubiquitin chains. Among them, only Lys-48-linked ubiquitin chains mediate proteasomal degradation^58^. Therefore, elucidating the exact HQQR-regulated ubiquitination sites on IP3R2 is conducive to exploring its potential pharmacological targets. In follow-up studies, we will combine mass spectrometry, site-directed mutagenesis, co-immunoprecipitation and ubiquitination functional assays to identify the HQQR-regulated ubiquitination sites on IP3R2 and characterize the biological functions of related ubiquitin ligases, so as to further elaborate the underlying regulatory mechanism governing MAM homeostasis.

The functional changes mediated by FUNDC1 are not consistent across different types of heart diseases. High glucose intake upregulates the expression of FUNDC1 and IP3R in cardiomyocytes. The increase in FUNDC1 promotes the formation of MAM, ultimately leading to mitochondrial Ca²⁺ overload, which is involved in diabetic cardiomyopathy^16^. Previously, we discovered that HQQR could improve mitochondrial biogenesis and ER stress^59^. This study found that HQQR reduced the expression of FUNDC1/IP3R2 in the myocardium of the OBH model, exerting myocardial protective effects. However, the disruption of the interaction between FUNDC1 and IP3R2 may also reduce the Ca²⁺ levels in mitochondria and cytosol, causing mitochondrial dysfunction, cardiac dysfunction, and heart failure^60^. Therefore, it is crucial to explore the dynamic changes of FUNDC1 and its roles in different heart disease models.

Our previous studies identified blood stasis, yang hyperactivity, and phlegm dampness as key pathological factors in OBH. HQQR, formulated according to traditional Chinese medicine principles, promotes blood circulation, suppresses yang, and resolves phlegm to eliminate dampness. Previous studies have demonstrated its effectiveness in lowering blood pressure, improving cardiac and renal function, reducing ER stress, and stabilizing mitochondrial activity^23,59^. In the present study, HQQR maintained mitochondrial Ca²⁺ homeostasis by regulating MAM, ameliorating myocardial remodeling while reducing blood pressure and obesity in OBH rats. FUNDC1 knockdown reduced IP3R2 expression, demonstrating the regulatory effect of FUNDC1 on IP3R2. This knockdown significantly downregulated FUNDC1 and IP3R2 expression, limiting excessive ER Ca²⁺ release and thus preventing mitochondrial Ca²⁺ overload. By attenuating mitochondrial Ca²⁺ accumulation and mitoROS generation, HQQR preserved mitochondrial function. Mitochondrial Ca²⁺ overload leads to excessive mitoROS production, which in turn induces oxidative stress and mitochondrial dysfunction. Furthermore, HQQR stabilized MAM structure, thereby maintaining mitochondrial Ca²⁺ homeostasis and energy metabolism. Collectively, HQQR mitigates OBH-induced myocardial remodeling primarily via modulation of FUNDC1/IP3R2-mediated Ca²⁺ transfer in MAM.

Clinically, OBH constitutes a prevalent high-risk phenotype characterized by poor cardiovascular outcomes resulting from combined metabolic and hemodynamic stress. Current therapeutic agents such as valsartan primarily target blood pressure reduction and neurohumoral activation through inhibition of the angiotensin II type 1 receptor (AT1R). Although valsartan has been reported to partially improve metabolic parameters, including insulin resistance and circulating lipid levels, these effects are generally limited and largely secondary to its hemodynamic actions^61^.Our findings suggest that HQQR exerts a better metabolic regulatory effect than valsartan. LW/BW serves as a robust and vital indicator of hepatic homeostasis, and its aberrance indicates pathological liver injury in metabolic-associated steatohepatitis models^62^. Higher hepatic fat content is strongly associated with elevated left ventricular mass index, suggesting a close link between hepatic steatosis and cardiac remodeling^63^.

Danshen, a key herb in HQQR and its active metabolites, also exerts beneficial effects in non-alcoholic fatty liver disease partly via regulating the PPARγ signaling pathway, which may partially account for the integrated metabolic and cardioprotective actions of HQQR^64^. Whether the liver-heart axis is involved in the protective effect of HQQR is worth further exploring. This multi-target regulatory capacity may explain its superior efficacy in improving both metabolic and cardiac outcomes compared with conventional Ang II receptor blocker therapy. These findings could inform future clinical management, particularly for hypertensive patients with comorbid obesity and metabolic syndrome.

As the core active constituents of Salvia miltiorrhiza (Danshen), salvianolic acid A^65,66^ and dihydrotanshinone I^67^ exert cardioprotective effects by scavenging mitoROS, stabilizing mitochondrial membrane potential, improving ATP generation, and relieving myocardial Ca²⁺ overload and apoptosis. Meanwhile, rhynchophylline^68,69^, isocorynoxeine^70^, and hirsutine^71^ exert vasodilatory effects to improve hypertension, and also attenuate myocardial oxidative stress and mitochondrial injury. Our network pharmacology study showed that HQQR might exert its effects by modulating mitochondrial membrane function, Ca²⁺ channel activity, and carboxylic acid metabolic processes. Therefore, Ca²⁺-mediated alterations in mitochondrial function may serve as a therapeutic target of HQQR.

MAM harbors abundant functional proteins^14^. From the perspective of traditional Chinese medicine theories, our study innovatively focused on FUNDC1/IP3R2- dependent Ca²⁺ homeostasis and ubiquitination modification at the MAM interface, thereby clarifying the molecular mechanism by which HQQR ameliorates OBH-induced myocardial remodeling. Nevertheless, this study has several inherent limitations. The lack of genetic knockout models limits causal inference regarding the FUNDC1/IP3R2 axis. MAM structure and function involve multiple protein complexes, including tethering proteins, yet the relationship between FUNDC1 and other MAM tethering complexes such as the IP3R-GRP75-VDAC complex remains incompletely characterized^72,73^. The regulatory mechanism of IP3R2 ubiquitination and its upstream regulatory factors have not been fully clarified. In addition, the lack of metabolism-targeted positive controls hinders the comprehensive evaluation of the metabolic regulatory efficacy of HQQR. Given the inherent poly-pharmacological characteristics of TCM formulas, subsequent studies will combine modern pharmacological techniques to further elucidate the molecular mechanisms responsible for the protective effects of HQQR. Additionally, all findings of the present study are derived from in vitro and animal experiments, with no verification in human samples. Further investigations utilizing clinical specimens and genetically modified models are therefore essential to validate and extend our current conclusions.

## Conclusions

**Fig. 6:**
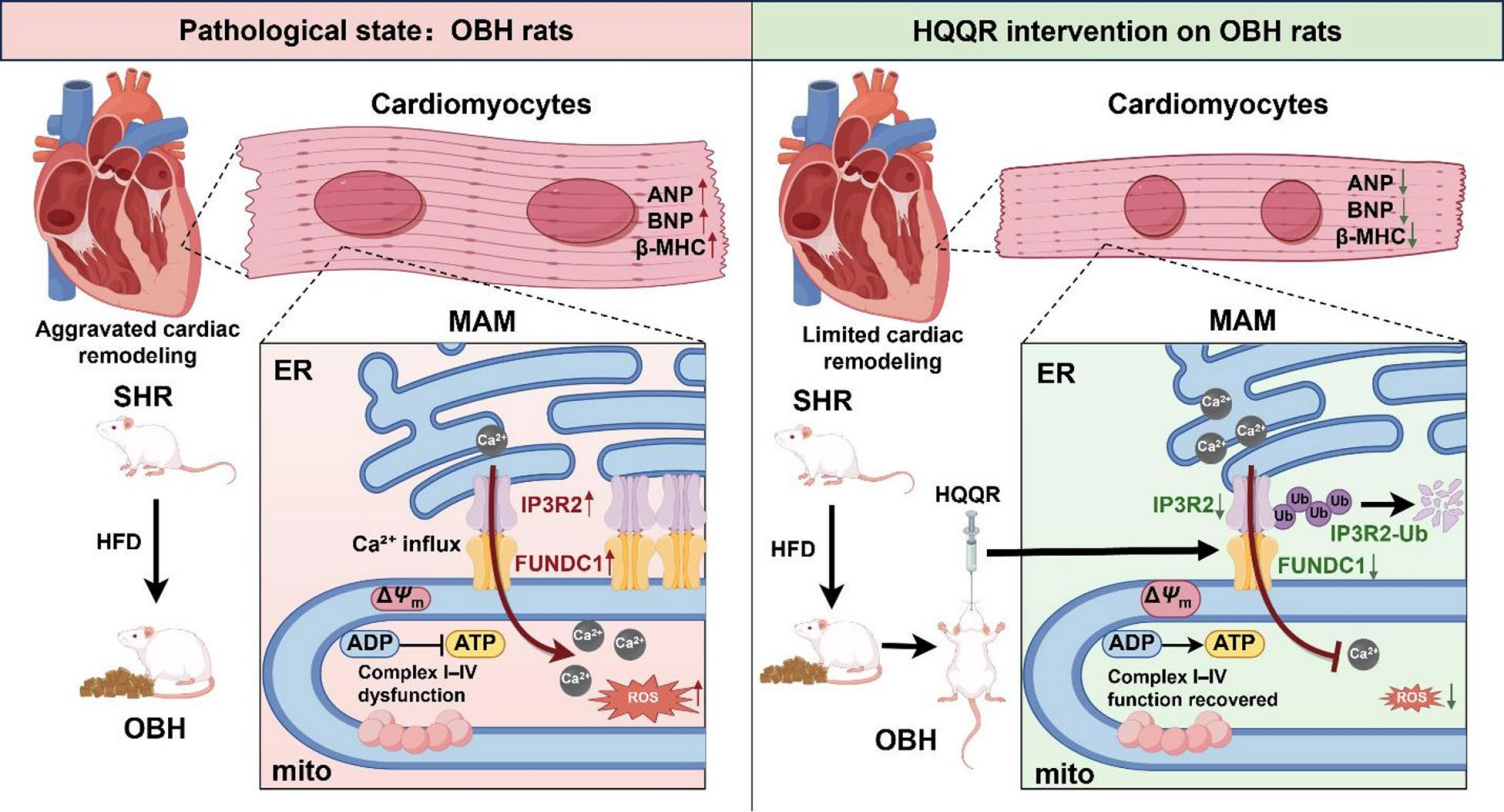
HQQR ameliorates myocardial injury in OBH rats via regulating FUNDC1/IP3R2‑mediated MAM function. Left panel: Under the pathological state of OBH, SHR fed a HFD develop aggravated cardiac remodeling, characterized by upregulated hypertrophy markers in cardiomyocytes. Increased expression of FUNDC1 and IP3R2 promotes excessive formation of MAMs, facilitating abnormal Ca²⁺ influx from the ER into mitochondria. This leads to mitochondrial Ca²⁺ overload and mitochondrial dysfunction, ultimately exacerbating myocardial damage. Right panel: HQQR downregulates FUNDC1 and IP3R2 expression and promotes ubiquitination-dependent degradation of IP3R2, thereby normalizing MAM structure and function. This reduces ER-to-mitochondria Ca²⁺ transfer, restores mitochondrial function, attenuates oxidative stress, and alleviates cardiac remodeling in OBH rats. *HQQR* Huoxue Qianyang Qutan Recipe; *OBH* obese hypertensive; *FUNDC1* FUN14 domain-containing protein 1; *IP3R2* inositol 1,4,5-trisphosphate receptor type 2; *MAM* mitochondria-associated endoplasmic reticulum membrane; *SHR* spontaneously hypertensive rats; *HFD* high-fat diet; *ANP* atrial natriuretic peptide; *BNP* B-type natriuretic peptide; *β-MHC* β-myosin heavy chain; *ER* endoplasmic reticulum; *mito* mitochondria; Δ*Ψ*_m_ mitochondrial membrane potential; *ATP* adenosine triphosphate; *ADP* adenosine diphosphate; *ROS* reactive oxygen species; *Ub* ubiquitin.

HQQR mitigates OBH-induced remodeling primarily via modulation of FUNDC1/IP3R2-mediated Ca²⁺ transfer at MAMs. These results support the potential use of HQQR in preventing and treating OBH while advancing our understanding of mitochondrial and Ca²⁺-targeted therapeutic approaches for cardiac remodeling (Fig. 6).

## Supplementary Information

Below is the link to the electronic supplementary material.

Supplementary Material 1

Supplementary Material 2

Supplementary Material 3

## Data Availability

The datasets used and/or analysed during the current study are available from the corresponding author on reasonable request.

## Abbreviations

OBH: Obese hypertension
MAM: Mitochondria-associated endoplasmic reticulum membrane
ER: Endoplasmic reticulum
mitoROS: Mitochondrial reactive oxygen species
mtDNA: mitochondrial DNA
IP3R2: Inositol 1,4,5-trisphosphate receptor type 2
FUNDC1: FUN14 domain-containing protein 1
HFD: High-fat diet
TCM: Traditional Chinese medicine
HQQR: Huoxue Qianyang Qutan Recipe
SHR: Spontaneously hypertensive rats
WKY: Wistar-Kyoto rats
ND: Normal diet
SBP: Systolic blood pressure
DBP: Diastolic blood pressure
MBP: Mean blood pressure
IVSD: Interventricular septal thickness
LVIDD: Left ventricular internal diameter at end-diastole
LVPWD: Left ventricular posterior wall thickness in diastole
EF: Ejection fraction
FS: Fractional shortening
LVM: Left ventricular mass
LVMI: Left ventricular mass index
HW/BW: Heart weight-to-body weight ratio
HW/TL: Heart weight-to-tibia length ratio
LW/BW: Liver weight -to- body weight ratio
H&E: Hematoxylin and eosin
WGA: Wheat germ agglutinin
HBSS: Hank’s Balanced Salt Solution
PBS: Phosphate Buffered Saline
Ang II: Angiotensin II
PVDF: Polyvinylidene difluoride
ANP: Atrial natriuretic peptide
BNP: Brain natriuretic peptide
β-MHC: β-myosin heavy chain
Ub: Ubiquitin
Co-IP: Co-immunoprecipitation
LSD: Least Significant Difference
ATP: Adenosine triphosphate
ELISA: Enzyme-linked immunosorbent assay
OD: Optical density
BMI: Body mass index
TEM: Transmission electron microscopy

## References

[1] Mills, K. T., Stefanescu, A. & He, J. The global epidemiology of hypertension. *Nat. Rev. Nephrol.* **16**, 223–237. 10.1038/s41581-019-0244-2 (2020). 32024986 10.1038/s41581-019-0244-2 PMC7998524

[2] Al-Makki, A. et al. Hypertension Pharmacological Treatment in Adults: A World Health Organization Guideline Executive Summary. *Hypertens. (Dallas Tex. : 1979)*. **79**, 293–301. 10.1161/HYPERTENSIONAHA.121.18192 (2022). 10.1161/HYPERTENSIONAHA.121.18192 PMC8654104 34775787

[3] Hall, J. E., Carmo, da Silva, J. M., Wang, A. A., Hall, M. E. & Z. & Obesity-induced hypertension: interaction of neurohumoral and renal mechanisms. *Circul. Res.* **116**, 991–1006. 10.1161/CIRCRESAHA.116.305697 (2015). 10.1161/CIRCRESAHA.116.305697 PMC4363087 25767285

[4] Bengmark, S. Obesity, the deadly quartet and the contribution of the neglected daily organ rest - a new dimension of un-health and its prevention. *Hepatobiliary Surg. Nutr.* **4**, 278–288. 10.3978/j.issn.2304-3881.2015.07.02 (2015). 26312244 10.3978/j.issn.2304-3881.2015.07.02 PMC4526765

[5] Yang, M. et al. Fibroblast growth factor 21 in metabolic syndrome. *Front. Endocrinol.* **14**, 1220426. 10.3389/fendo.2023.1220426 (2023). 10.3389/fendo.2023.1220426 PMC10414186 37576954

[6] Du, W. et al. Plasma levels of heart failure biomarkers are primarily a reflection of extracardiac production. *Theranostics* **8**, 4155–4169. 10.7150/thno.26055 (2018). 30128044 10.7150/thno.26055 PMC6096401

[7] Tuccinardi, D. et al. Rethinking weight loss treatments as cardiovascular medicine in obesity, a comprehensive review. *Eur. J. Prev. Cardiol.*10.1093/eurjpc/zwae171 (2024). 38833329 10.1093/eurjpc/zwae171

[8] Zhang, S. et al. Effect and mechanism of Qing Gan Zi Shen decoction on heart damage induced by obesity and hypertension. *J. Ethnopharmacol.* **319**, 117163. 10.1016/j.jep.2023.117163 (2024). 37741474 10.1016/j.jep.2023.117163

[9] Gui, M. et al. Huoxue Qianyang Qutan recipe attenuates Ang II-induced cardiomyocyte hypertrophy by regulating reactive oxygen species production. *Experimental therapeutic Med.* **22**, 1446. 10.3892/etm.2021.10881 (2021). 10.3892/etm.2021.10881 PMC8549094 34721688

[10] Szymański, J. et al. Interaction of Mitochondria with the Endoplasmic Reticulum and Plasma Membrane in Calcium Homeostasis, Lipid Trafficking and Mitochondrial Structure. *Int. J. Mol. Sci.* **18**10.3390/ijms18071576 (2017). 10.3390/ijms18071576 PMC5536064 28726733

[11] de Brito, O. M. & Scorrano, L. Mitofusin-2 regulates mitochondrial and endoplasmic reticulum morphology and tethering: the role of Ras. *Mitochondrion* **9**, 222–226. 10.1016/j.mito.2009.02.005 (2009). 19269351 10.1016/j.mito.2009.02.005

[12] D’Eletto, M. et al. Transglutaminase Type 2 Regulates ER-Mitochondria Contact Sites by Interacting with GRP75. *Cell. Rep.* **25**, 3573–3581. 10.1016/j.celrep.2018.11.094 (2018). e3574. 30590033 10.1016/j.celrep.2018.11.094

[13] Li, Z. et al. The SIRT3-ATAD3A axis regulates MAM dynamics and mitochondrial calcium homeostasis in cardiac hypertrophy. *Int. J. Biol. Sci.* **20**, 831–847. 10.7150/ijbs.89253 (2024). 38250153 10.7150/ijbs.89253 PMC10797690

[14] Lu, B. et al. So close, yet so far away: the relationship between MAM and cardiac disease. *Front. Cardiovasc. Med.* **11**, 1353533. 10.3389/fcvm.2024.1353533 (2024). 38374992 10.3389/fcvm.2024.1353533 PMC10875081

[15] Wu, S. et al. Binding of FUN14 Domain Containing 1 With Inositol 1,4,5-Trisphosphate Receptor in Mitochondria-Associated Endoplasmic Reticulum Membranes Maintains Mitochondrial Dynamics and Function in Hearts in Vivo. *Circulation* **136**, 2248–2266. 10.1161/CIRCULATIONAHA.117.030235 (2017). 28942427 10.1161/CIRCULATIONAHA.117.030235 PMC5716911

[16] Wu, S. et al. Hyperglycemia-Driven Inhibition of AMP-Activated Protein Kinase alpha2 Induces Diabetic Cardiomyopathy by Promoting Mitochondria-Associated Endoplasmic Reticulum Membranes In Vivo. *Circulation* **139**, 1913–1936. 10.1161/CIRCULATIONAHA.118.033552 (2019). 30646747 10.1161/CIRCULATIONAHA.118.033552 PMC6465113

[17] Wang, C. et al. FUNDC1-dependent mitochondria-associated endoplasmic reticulum membranes are involved in angiogenesis and neoangiogenesis. *Nat. Commun.* **12**, 2616. 10.1038/s41467-021-22771-3 (2021). 33972548 10.1038/s41467-021-22771-3 PMC8110587

[18] Ren, J. et al. FUNDC1 interacts with FBXL2 to govern mitochondrial integrity and cardiac function through an IP3R3-dependent manner in obesity. *Sci. Adv.* **6**10.1126/sciadv.abc8561 (2020). 10.1126/sciadv.abc8561 PMC7494344 32938669

[19] Tan, C. et al. Puerarin Improves Vascular Insulin Resistance and Cardiovascular Remodeling in Salt-Sensitive Hypertension. *Am. J. Chin. Med.* **45**, 1169–1184. 10.1142/S0192415X17500641 (2017). 28830209 10.1142/S0192415X17500641

[20] Wang, P., Hao, D. & Xiong, X. Anti-hypertension effect of Wuwei Jiangya decoction via ACE2/Ang1-7/MAS signaling pathway in SHR based on network degree-distribution analysis. *J. Ethnopharmacol.* **319**, 117121. 10.1016/j.jep.2023.117121 (2024). 37660954 10.1016/j.jep.2023.117121

[21] Xie, J. et al. Efficacy of Huoxue Qianyang Qutan Recipe on essential hypertension: A randomized, double-blind, placebo-controlled trial. *J. Integr. Med.* **22**, 484–492. 10.1016/j.joim.2024.05.002 (2024). 38789290 10.1016/j.joim.2024.05.002

[22] Li, G., Li, J., Shao, R., Zhao, J. & Chen, M. FUNDC1: A Promising Mitophagy Regulator at the Mitochondria-Associated Membrane for Cardiovascular Diseases. *Front. cell. Dev. biology*. **9**, 788634. 10.3389/fcell.2021.788634 (2021). 10.3389/fcell.2021.788634 PMC8797154 35096821

[23] Lu, B. et al. Huoxue Qianyang Qutan recipe attenuates cardiac fibrosis by inhibiting the NLRP3 inflammasome signalling pathway in obese hypertensive rats. *Pharm. Biol.* **59**, 1045–1057. 10.1080/13880209.2021.1953541 (2021). 34362291 10.1080/13880209.2021.1953541 PMC8354174

[24] Wang, J. et al. HuoXue QianYang QuTan Recipe attenuates left ventricular hypertrophy in obese hypertensive rats by improving mitochondrial function through SIRT1/PGC-1α deacetylation pathway. *Biosci. Rep.* **39**10.1042/bsr20192909 (2019). 10.1042/BSR20192909 PMC6923340 31778153

[25] Wang, M. et al. Huoxue Qianyang Qutan Recipe protects against early renal damage induced by obesity-related hypertension via the SIRT1/NF-κB/IL-6 Pathway: Integrating network pharmacology and experimental validation-based strategy. *Evid. Based Complement. Altern. Med. eCAM* 9599090 (2022). 10.1155/2022/9599090 10.1155/2022/9599090 PMC9166942 35668772

[26] Levin, B. E. Arcuate NPY neurons and energy homeostasis in diet-induced obese and resistant rats. *Am. J. Physiol.* **276**, R382–387. 10.1152/ajpregu.1999.276.2.R382 (1999). 9950915 10.1152/ajpregu.1999.276.2.R382

[27] Zhang, P. et al. Alterations to the microbiota-colon-brain axis in high-fat-diet-induced obese mice compared to diet-resistant mice. *J. Nutr. Biochem.* **65**, 54–65. 10.1016/j.jnutbio.2018.08.016 (2019). 30623851 10.1016/j.jnutbio.2018.08.016

[28] Lee, S., Jang, S., Kim, J. Y. & Kim, I. Dahl Salt-Resistant Rat Is Protected against Hypertension during Diet-Induced Obesity. *Nutrients* **14**10.3390/nu14183843 (2022). 10.3390/nu14183843 PMC9506364 36145220

[29] Sacks, D. et al. Multisociety Consensus Quality Improvement Revised Consensus Statement for Endovascular Therapy of Acute Ischemic Stroke. *Int. J. Stroke*. **13**, 612–632. 10.1177/1747493018778713 (2018). 29786478 10.1177/1747493018778713

[30] Piazza, M. et al. Anatomic Feasibility of a Specifically Designed Custom Made Device as Off the Shelf Endograft for Urgent Repair of Complex Abdominal Aortic Aneurysm. *Eur. J. Vasc Endovasc Surg.*10.1016/j.ejvs.2025.12.009 (2025). 41380764 10.1016/j.ejvs.2025.12.009

[31] Zhang, X. et al. Valsartan regulates myocardial autophagy and mitochondrial turnover in experimental hypertension. *Hypertension (Dallas, Tex.* **64**, 87–93. 10.1161/hypertensionaha.113.02151 (2014). 10.1161/HYPERTENSIONAHA.113.02151 PMC4057304 24752430

[32] Ru, J. et al. TCMSP: a database of systems pharmacology for drug discovery from herbal medicines. *J. Cheminform*. **6**, 13. 10.1186/1758-2946-6-13 (2014). 24735618 10.1186/1758-2946-6-13 PMC4001360

[33] Szklarczyk, D. et al. STITCH 5: augmenting protein-chemical interaction networks with tissue and affinity data. *Nucleic Acids Res.* **44**, D380–384. 10.1093/nar/gkv1277 (2016). 26590256 10.1093/nar/gkv1277 PMC4702904

[34] Kim, S. et al. PubChem in 2021: new data content and improved web interfaces. *Nucleic Acids Res.* **49**, D1388–d1395. 10.1093/nar/gkaa971 (2021). 33151290 10.1093/nar/gkaa971 PMC7778930

[35] UniProt. a worldwide hub of protein knowledge. *Nucleic Acids Res.* **47**, D506–d515. 10.1093/nar/gky1049 (2019). 30395287 10.1093/nar/gky1049 PMC6323992

[36] Stelzer, G. et al. The GeneCards Suite: From Gene Data Mining to Disease Genome Sequence Analyses. *Curr. Protoc. Bioinf.* **54**, 1.30.31–31.30.33 (2016). 10.1002/cpbi.5 27322403

[37] Piñero, J. et al. DisGeNET: a comprehensive platform integrating information on human disease-associated genes and variants. *Nucleic Acids Res.* **45**, D833–d839. 10.1093/nar/gkw943 (2017). 27924018 10.1093/nar/gkw943 PMC5210640

[38] Hariri, N. & Thibault, L. High-fat diet-induced obesity in animal models. *Nutr. Res. Rev.* **23**, 270–299. 10.1017/s0954422410000168 (2010). 20977819 10.1017/S0954422410000168

[39] Gomez, L. et al. The SR/ER-mitochondria calcium crosstalk is regulated by GSK3beta during reperfusion injury. *Cell Death Differ.* **23**, 313–322. 10.1038/cdd.2015.101 (2016). 26206086 10.1038/cdd.2015.101 PMC4716295

[40] Bardou, P., Mariette, J., Escudié, F., Djemiel, C. & Klopp, C. jvenn: an interactive Venn diagram viewer. *BMC Bioinform.* **15**, 293. 10.1186/1471-2105-15-293 (2014). 10.1186/1471-2105-15-293 PMC4261873 25176396

[41] Sun, N. & Finkel, T. Cardiac mitochondria: a surprise about size. *J. Mol. Cell. Cardiol.* **82**, 213–215. 10.1016/j.yjmcc.2015.01.009 (2015). 25626176 10.1016/j.yjmcc.2015.01.009 PMC8760902

[42] Akhmedov, A. T., Rybin, V. & Marin-Garcia, J. Mitochondrial oxidative metabolism and uncoupling proteins in the failing heart. *Heart Fail. Rev.* **20**, 227–249. 10.1007/s10741-014-9457-4 (2015). 25192828 10.1007/s10741-014-9457-4

[43] Dia, M. et al. Effect of Metformin on T2D-Induced MAM Ca2 + Uncoupling and Contractile Dysfunction in an Early Mouse Model of Diabetic HFpEF. *Int. J. Mol. Sci.* **23**10.3390/ijms23073569 (2022). 10.3390/ijms23073569 PMC8998623 35408928

[44] Osanami, A. et al. Adenosine monophosphate deaminase in the endoplasmic reticulum-mitochondria interface promotes mitochondrial Ca2 + overload in type 2 diabetes rat hearts. *J. diabetes Invest.* **14**, 560–569. 10.1111/jdi.13982 (2023). 10.1111/jdi.13982 PMC10034956 36815317

[45] Karlstad, J., Sun, Y. & Singh, B. B. Ca(2+) signaling: an outlook on the characterization of Ca(2+) channels and their importance in cellular functions. *Adv. Exp. Med. Biol.* **740**, 143–157. 10.1007/978-94-007-2888-2_6 (2012). 22453941 10.1007/978-94-007-2888-2_6 PMC3316125

[46] Srikanth, S., Woo, J. S., Sun, Z. & Gwack, Y. Immunological Disorders: Regulation of Ca2 + Signaling in T Lymphocytes. *Adv. Exp. Med. Biol.* **993**, 397–424. 10.1007/978-3-319-57732-6_21 (2017). 28900926 10.1007/978-3-319-57732-6_21 PMC8957800

[47] Zhang, Y., Ye, L., Duan, D. D., Yang, H. & Ma, T. TMEM16A Plays an Insignificant Role in Myocardium Remodeling but May Promote Angiogenesis of Heart During Pressure-overload. *Front. Physiol.* **13**, 897619. 10.3389/fphys.2022.897619 (2022). 35711304 10.3389/fphys.2022.897619 PMC9194855

[48] Abudureyimu, M. et al. Berberine alleviates myocardial diastolic dysfunction by modulating Drp1-mediated mitochondrial fission and Ca(2+) homeostasis in a murine model of HFpEF. *Front. Med.* **17**, 1219–1235. 10.1007/s11684-023-0983-0 (2023). 37656418 10.1007/s11684-023-0983-0

[49] Gao, P., Yan, Z. & Zhu, Z. Mitochondria-Associated Endoplasmic Reticulum Membranes in Cardiovascular Diseases. *Front. cell. Dev. biology*. **8**, 604240. 10.3389/fcell.2020.604240 (2020). 10.3389/fcell.2020.604240 PMC7680862 33240899

[50] Gong, Y. et al. Mitochondria-associated membrane-modulated Ca2 + transfer: A potential treatment target in cardiac ischemia reperfusion injury and heart failure. *Life Sci.* **278**, 119511. 10.1016/j.lfs.2021.119511 (2021). 33864818 10.1016/j.lfs.2021.119511

[51] Chen, Q. et al. Endoplasmic reticulum stress and mitochondrial dysfunction during aging: Role of sphingolipids. *Biochim. et Biophys. acta Mol. cell. biology lipids*. **1868**, 159366. 10.1016/j.bbalip.2023.159366 (2023). 10.1016/j.bbalip.2023.159366 PMC11154090 37473835

[52] Frey, N., McKinsey, T. A. & Olson, E. N. Decoding calcium signals involved in cardiac growth and function. *Nat. Med.* **6**, 1221–1227. 10.1038/81321 (2000). 11062532 10.1038/81321

[53] Tian, S. et al. Sulforaphane Balances Ca2 + Homeostasis injured by excessive fat via mitochondria-associated membrane (MAM). *Mol. Nutri. Food Res.* **65**, e2001076. 10.1002/mnfr.202001076 (2021). 10.1002/mnfr.202001076 33929090

[54] Chen, W. et al. Polygonatum sibiricum polysaccharide ameliorates skeletal muscle aging via mitochondria-associated membrane-mediated calcium homeostasis regulation. *Phytomedicine*. **129**, 155567. 10.1016/j.phymed.2024.155567 (2024). 10.1016/j.phymed.2024.155567 38579644

[55] Luan, Y. et al. Structure and function of mitochondria-associated endoplasmic reticulum membranes (MAMs) and their role in cardiovascular diseases. *Oxid. Med. Cell. Longev.* **2021**, 4578809. 10.1155/2021/4578809 (2021). 10.1155/2021/4578809 PMC8289621 34336092

[56] Bartok, A. et al. IP3 receptor isoforms differently regulate ER-mitochondrial contacts and local calcium transfer. *Nat. Commun.* **10**, 3726. 10.1038/s41467-019-11646-3 (2019). 31427578 10.1038/s41467-019-11646-3 PMC6700175

[57] Grunfeld, C. et al. Contribution of metabolic and anthropometric abnormalities to cardiovascular disease risk factors. *Circulation* **118**, e20–28. 10.1161/circulationaha.107.189623 (2008). 18566314 10.1161/CIRCULATIONAHA.107.189623 PMC3170411

[58] Sliter, D. A., Aguiar, M., Gygi, S. P. & Wojcikiewicz, R. J. Activated inositol 1,4,5-trisphosphate receptors are modified by homogeneous Lys-48- and Lys-63-linked ubiquitin chains, but only Lys-48-linked chains are required for degradation. *J. Biol. Chem.* **286**, 1074–1082. 10.1074/jbc.M110.188383 (2011). 21071436 10.1074/jbc.M110.188383 PMC3020714

[59] Zhou, X. et al. Huoxue Qianyang decoction ameliorates cardiac remodeling in obese spontaneously hypertensive rats in association with ATF6-CHOP endoplasmic reticulum stress signaling pathway regulation. *Biomed. pharmacotherapy = Biomedecine pharmacotherapie*. **121**, 109518. 10.1016/j.biopha.2019.109518 (2020). 31689600 10.1016/j.biopha.2019.109518

[60] Wu, S. et al. Hyperglycemia-Driven Inhibition of AMP-Activated Protein Kinase α2 Induces Diabetic Cardiomyopathy by Promoting Mitochondria-Associated Endoplasmic Reticulum Membranes In Vivo. *Circulation* **139**, 1913–1936. 10.1161/circulationaha.118.033552 (2019). 30646747 10.1161/CIRCULATIONAHA.118.033552 PMC6465113

[61] Oliveira-Junior, S. A. et al. AT1 receptor blockade attenuates insulin resistance and myocardial remodeling in rats with diet-induced obesity. *PLoS One*. **9**, e86447. 10.1371/journal.pone.0086447 (2014). 24466104 10.1371/journal.pone.0086447 PMC3900554

[62] Yang, C. R. et al. Phenotypic and metabolomic characteristics of mouse models of metabolic associated steatohepatitis. *Biomark. Res.* **12**, 6. 10.1186/s40364-023-00555-9 (2024). 38195587 10.1186/s40364-023-00555-9 PMC10777576

[63] Kostka, F. et al. Cardiac remodelling in non-alcoholic fatty liver disease in the general population. *Liver Int.*10.1111/liv.15844 (2024). 38293745 10.1111/liv.15844

[64] Liu, J. et al. Salvia miltiorrhiza Bge. (Danshen) in the Treating Non-alcoholic Fatty Liver Disease Based on the Regulator of Metabolic Targets. *Front. Cardiovasc. Med.* **9**, 842980. 10.3389/fcvm.2022.842980 (2022). 35528835 10.3389/fcvm.2022.842980 PMC9072665

[65] Zhang, J. Y. et al. Salvianolic Acid A Ameliorates Arsenic Trioxide-Induced Cardiotoxicity Through Decreasing Cardiac Mitochondrial Injury and Promotes Its Anticancer Activity. *Front. Pharmacol.* **9**, 487. 10.3389/fphar.2018.00487 (2018). 29867492 10.3389/fphar.2018.00487 PMC5954107

[66] Li, X. L., Fan, J. P., Liu, J. X. & Liang, L. N. Salvianolic Acid A Protects Neonatal Cardiomyocytes Against Hypoxia/Reoxygenation-Induced Injury by Preserving Mitochondrial Function and Activating Akt/GSK-3β Signals. *Chin. J. Integr. Med.* **25**, 23–30. 10.1007/s11655-016-2747-z (2019). 28197936 10.1007/s11655-016-2747-z

[67] Wu, C. et al. 16-Dihydrotanshinone I protects against ischemic stroke by inhibiting ferroptosis via the activation of nuclear factor erythroid 2-related factor 2. *Phytomedicine*. **15**, 154790. 10.1016/j.phymed.2023.154790 (2023). 10.1016/j.phymed.2023.154790 37028247

[68] Qin, Q. J. et al. Rhynchophylline ameliorates myocardial ischemia/reperfusion injury through the modulation of mitochondrial mechanisms to mediate myocardial apoptosis. *Mol. Med. Rep.* **19**, 2581–2590. 10.3892/mmr.2019.9908 (2019). 30720139 10.3892/mmr.2019.9908 PMC6423601

[69] Lin, L. et al. Rhynchophylline as an agonist of sirtuin 3 ameliorates endothelial dysfunction via antagonizing mitochondrial damage of endothelial progenitor cells. *Br. J. Pharmacol.*10.1111/bph.70032 (2025). 40164963 10.1111/bph.70032

[70] Zhang, W. B., Chen, C. X., Sim, S. M. & Kwan, C. Y. In vitro vasodilator mechanisms of the indole alkaloids rhynchophylline and isorhynchophylline, isolated from the hook of Uncaria rhynchophylla (Miquel). *Naunyn Schmiedebergs Arch. Pharmacol.* **369**, 232–238. 10.1007/s00210-003-0854-9 (2004). 14668978 10.1007/s00210-003-0854-9

[71] Jiang, W. et al. Hirsutine ameliorates myocardial ischemia-reperfusion injury through improving mitochondrial function via CaMKII pathway. *Clin. Exp. Hypertens.* **45**, 2192444. 10.1080/10641963.2023.2192444 (2023). 36951068 10.1080/10641963.2023.2192444

[72] Mogk, A. & Bukau, B. Mitochondria tether protein trash to rejuvenate cellular environments. *Cell* **159**, 471–472. 10.1016/j.cell.2014.10.007 (2014). 25417098 10.1016/j.cell.2014.10.007

[73] Szabadkai, G. et al. Chaperone-mediated coupling of endoplasmic reticulum and mitochondrial Ca2 + channels. *J. Cell Biol.* **175**, 901–911. 10.1083/jcb.200608073 (2006). 17178908 10.1083/jcb.200608073 PMC2064700

