# Supplementary material for: Huoxue Qianyang Qutan Recipe limits cardiac remodeling by regulating FUNDC1/IP3R2 signaling pathway in obese hypertensive rats
Source: Sci Rep. 2026 Jun 4;16:25449. doi: 10.1038/s41598-026-56197-y (PMC13473496; doi:10.1038/s41598-026-56197-y)

# Original Blot Figure 1

## Unedited Immunoblots

**Full unedited blot for Figure 3A：FUNDC1 17kDa**

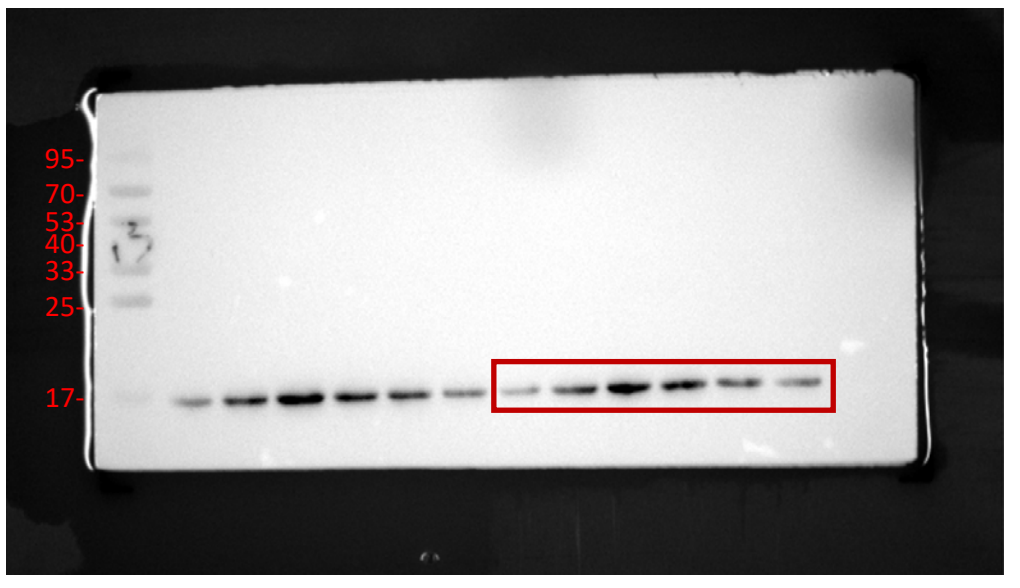


**Full unedited blot for Figure 3A：IP3R2 308kDa**

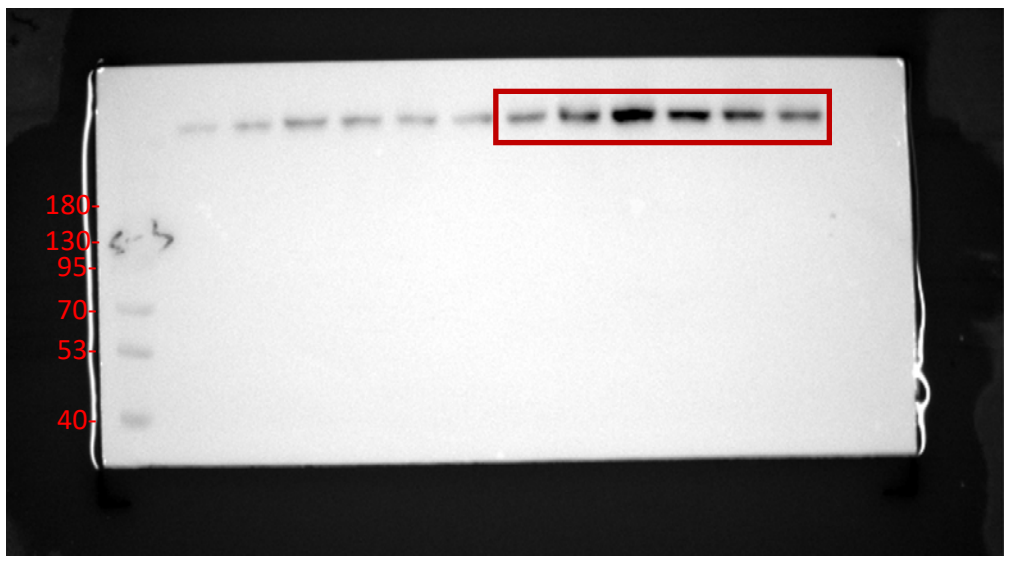


**Full unedited blot for Figure 3A：GAPDH 37kDa**

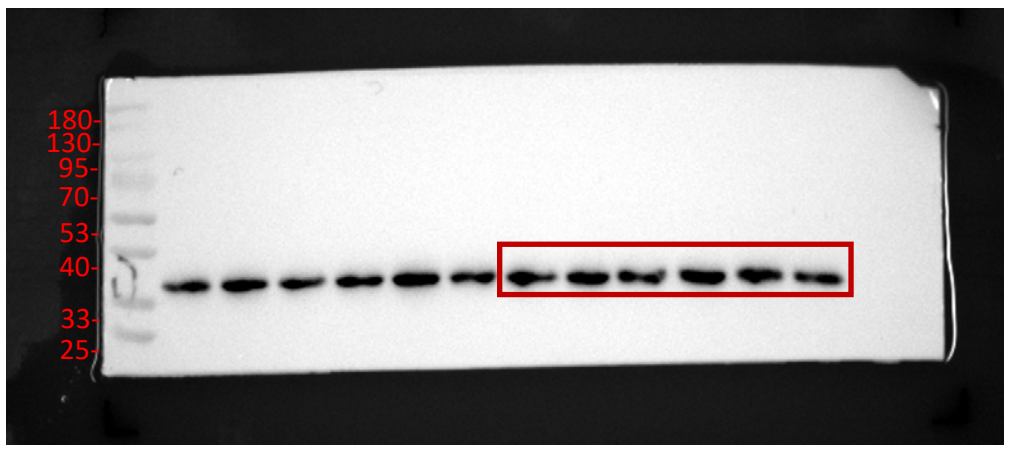

## Original Blot Figure 2

## Unedited Immunoblots

**Full unedited blot for Figure 3B：FUNDC1 17kDa**

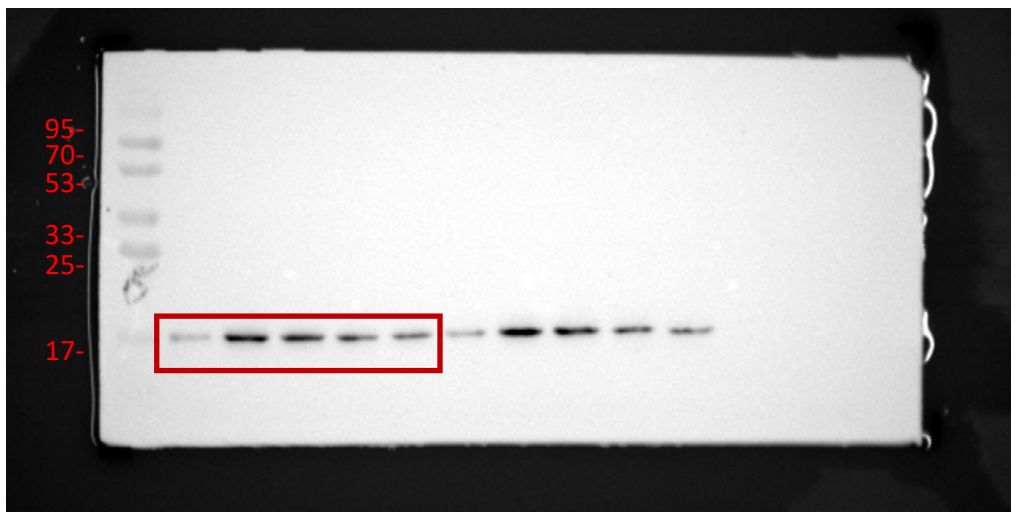


**Full unedited blot for Figure 3B：IP3R2 308kDa**

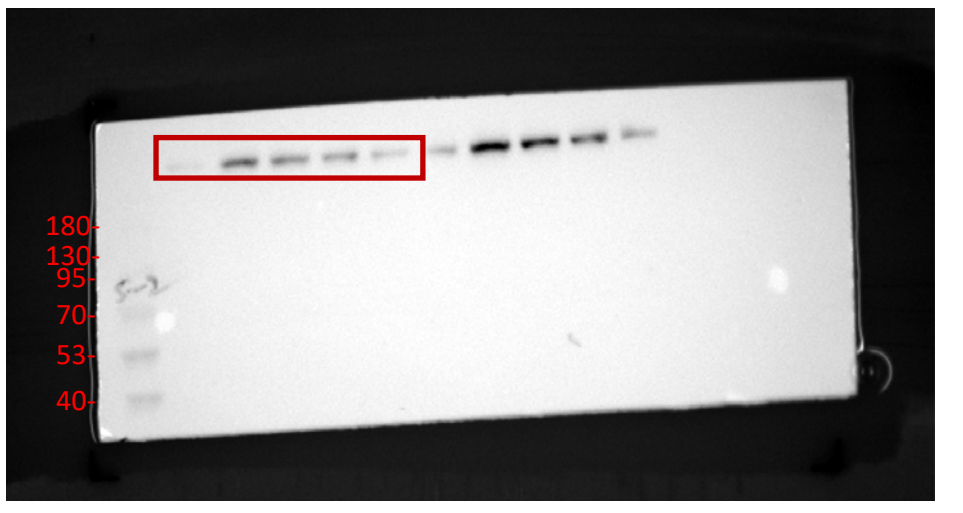


**Full unedited blot for Figure 3B：Calreticulin 48kDa**

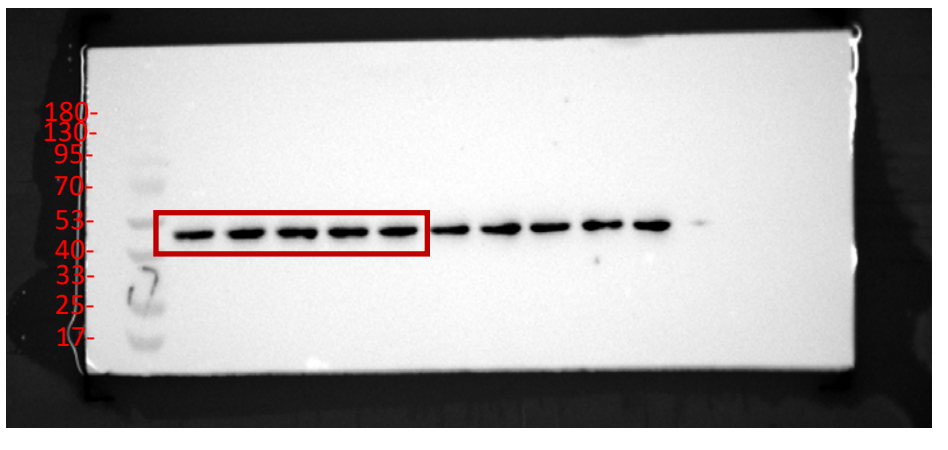

# Original Blot Figure 3

## Unedited Immunoblots

**Full unedited blot for Figure 3D：FUNDC1 17kDa**

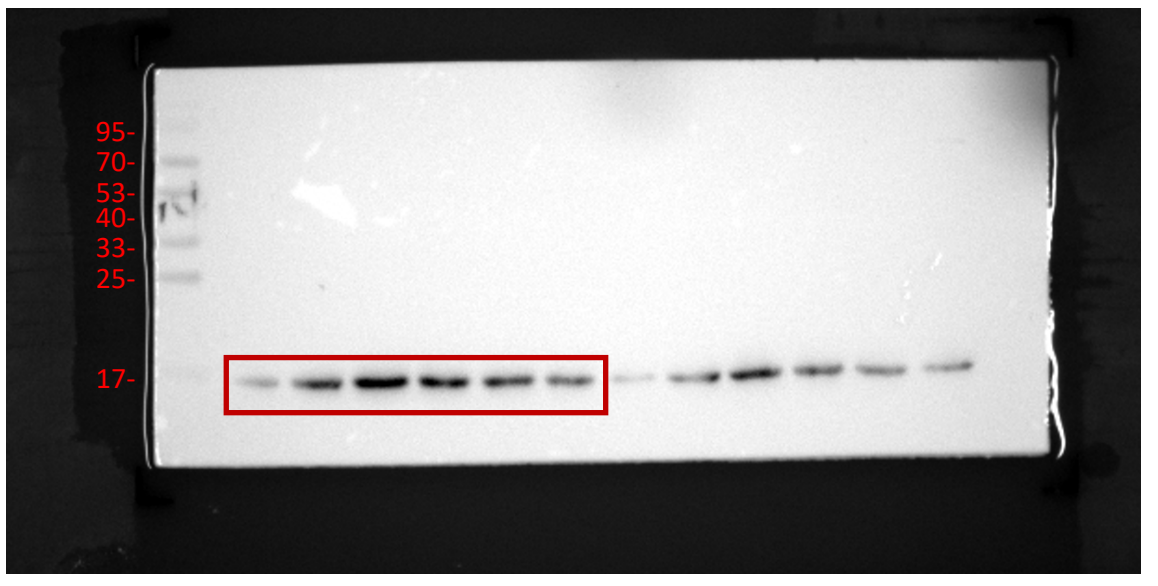


**Full unedited blot for Figure 3D：IP3R2 308kDa**

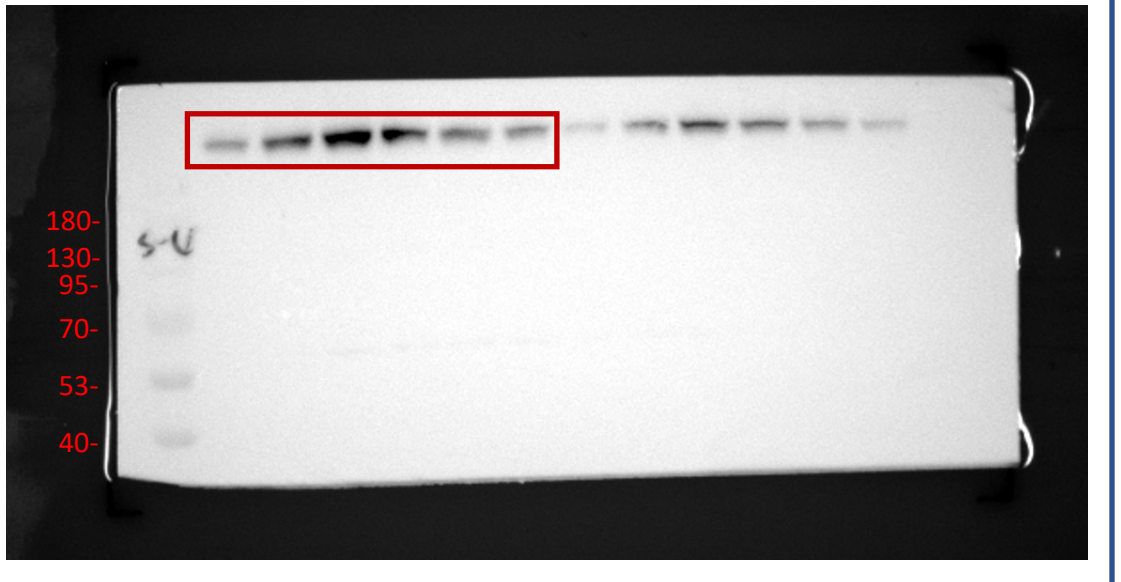


**Full unedited blot for Figure 3D：GAPDH 37kDa**

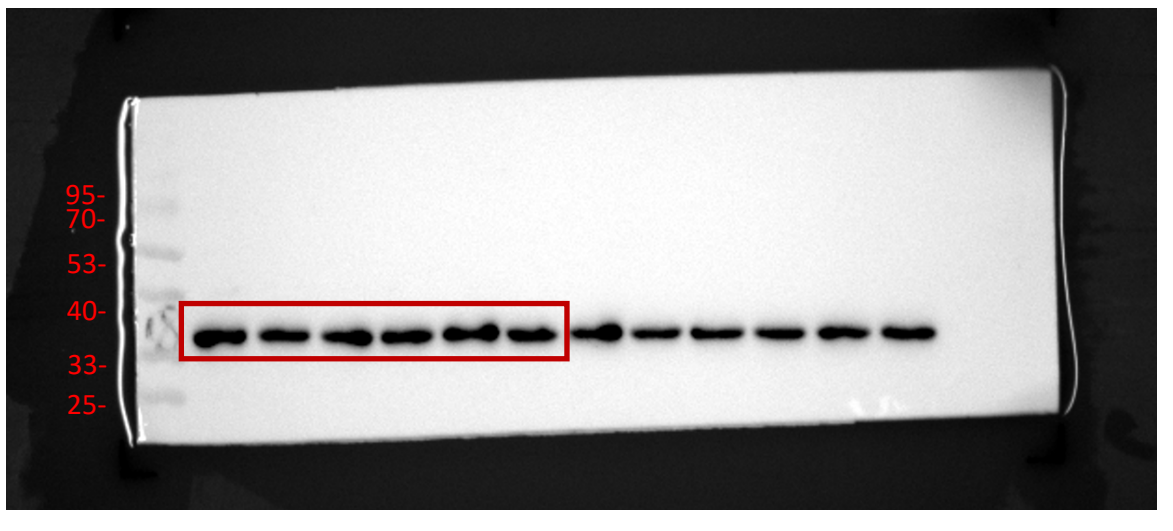


**Full unedited blot for Figure 3D：IP3R2 308kDa**

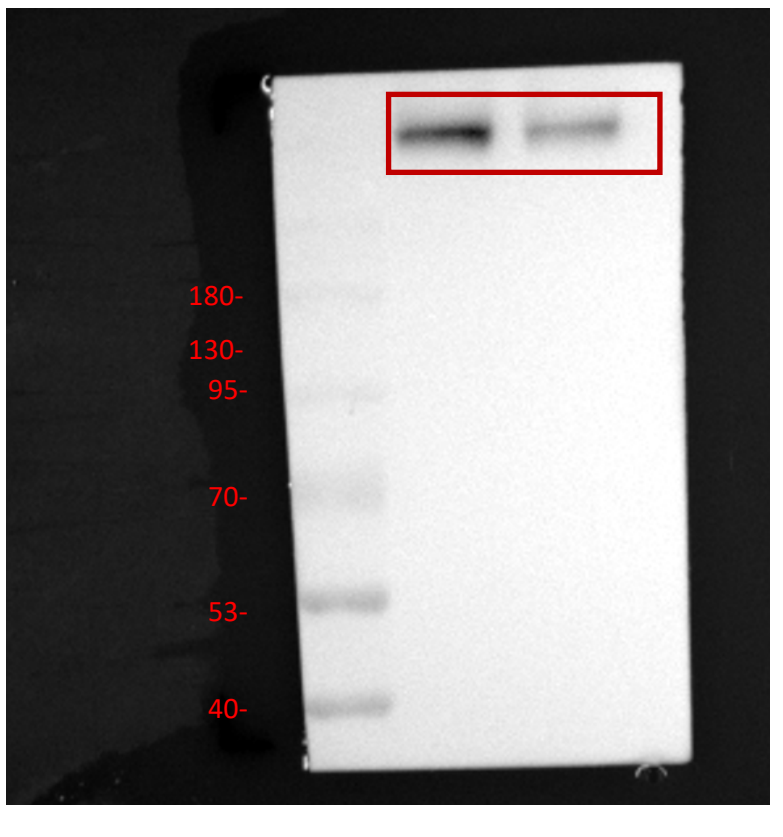


**Full unedited blot for Figure 3D：GAPDH 37kDa**

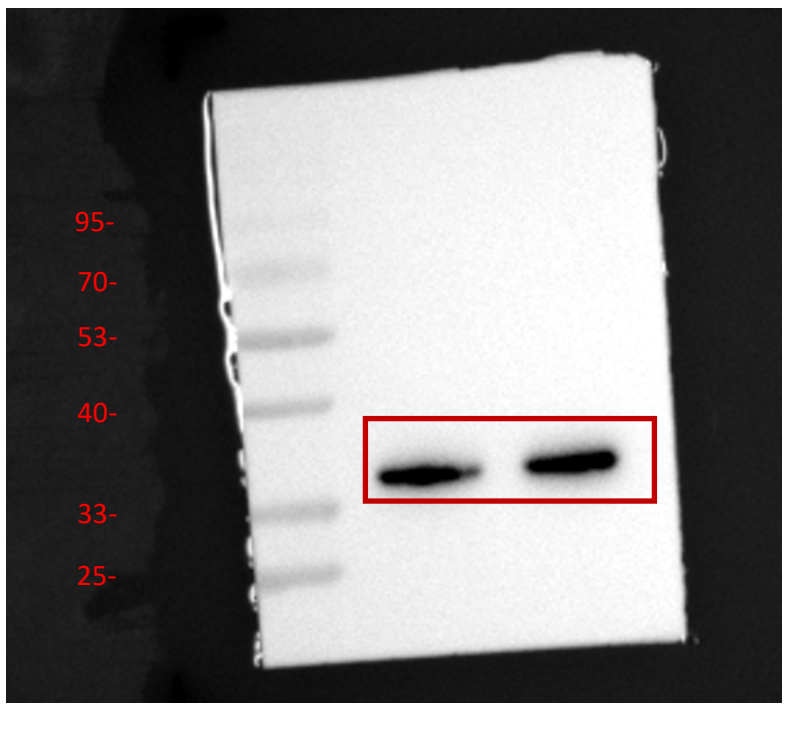


**Full unedited blot for Figure 3D and 3E：ubiquitination of IP3R2**

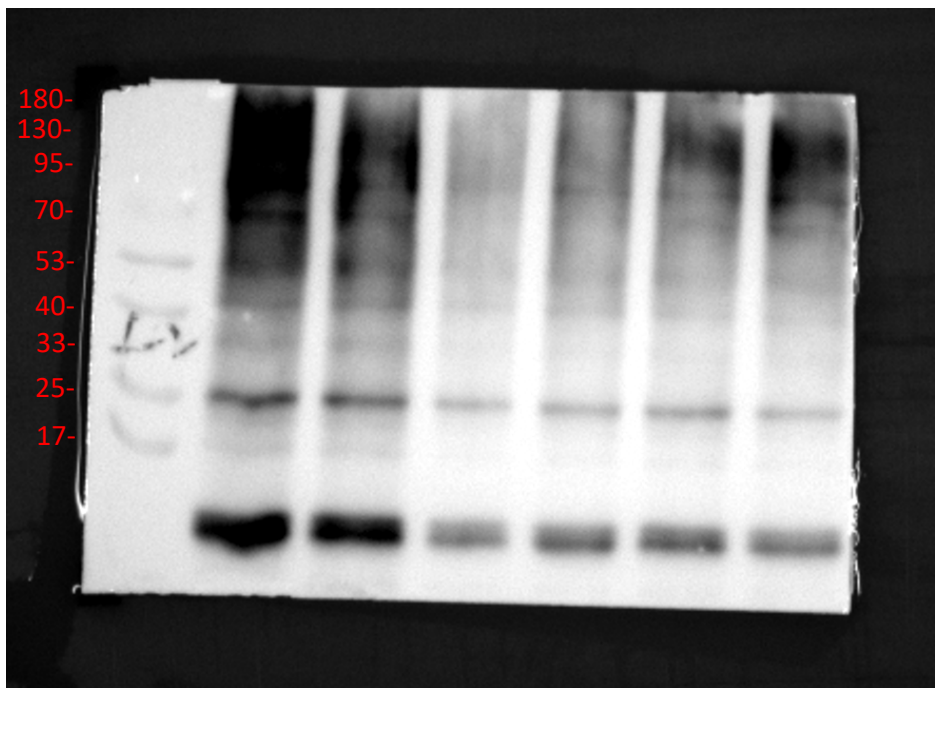


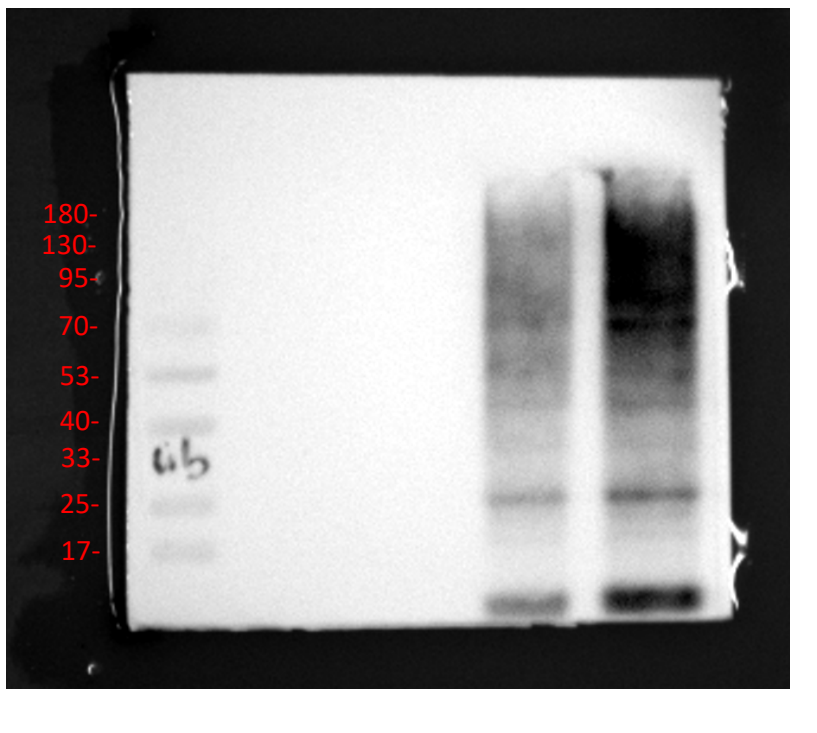

# Original Blot Figure 4

## Unedited Immunoblots

**Full unedited blot for Figure 4A:　ANP 17kDa**

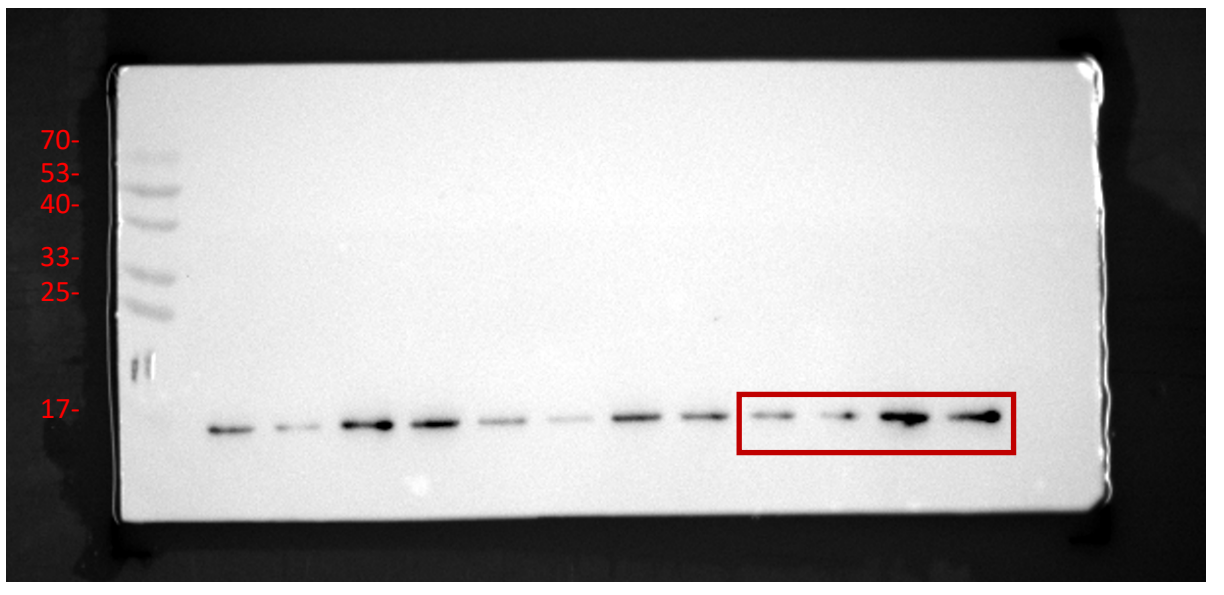


**Full unedited blot for Figure 4A:　BNP 15kDa**

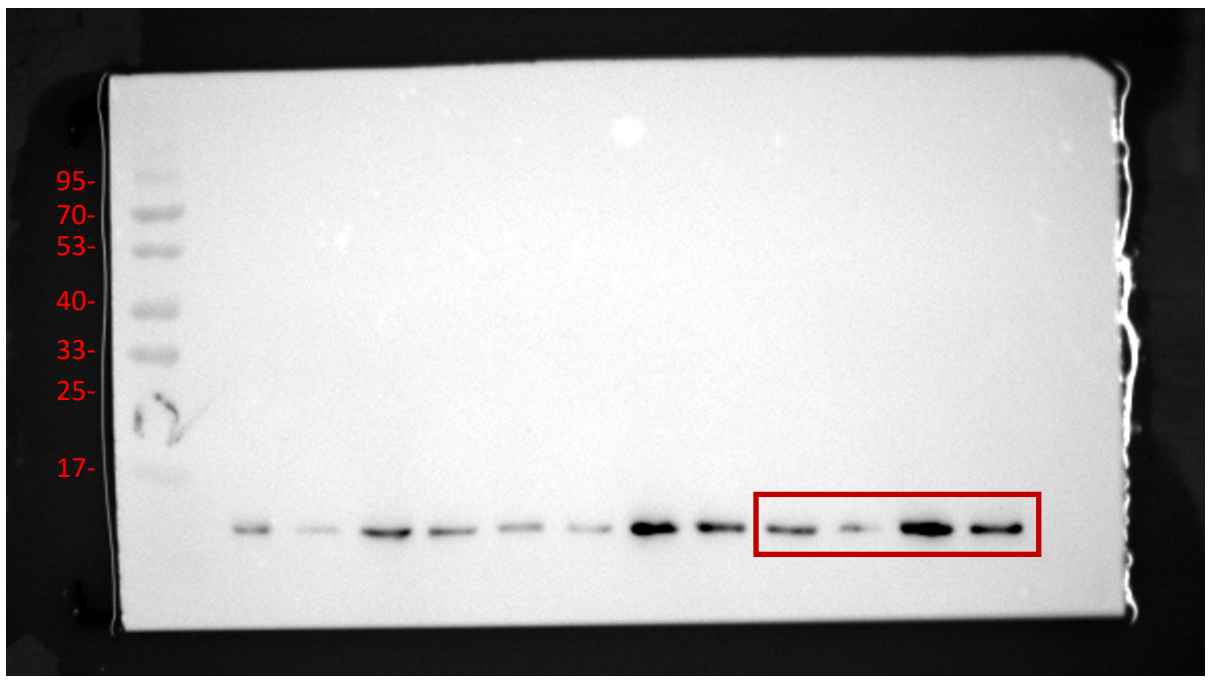


**Full unedited blot for Figure 4A:　β-MHC 220kDa**

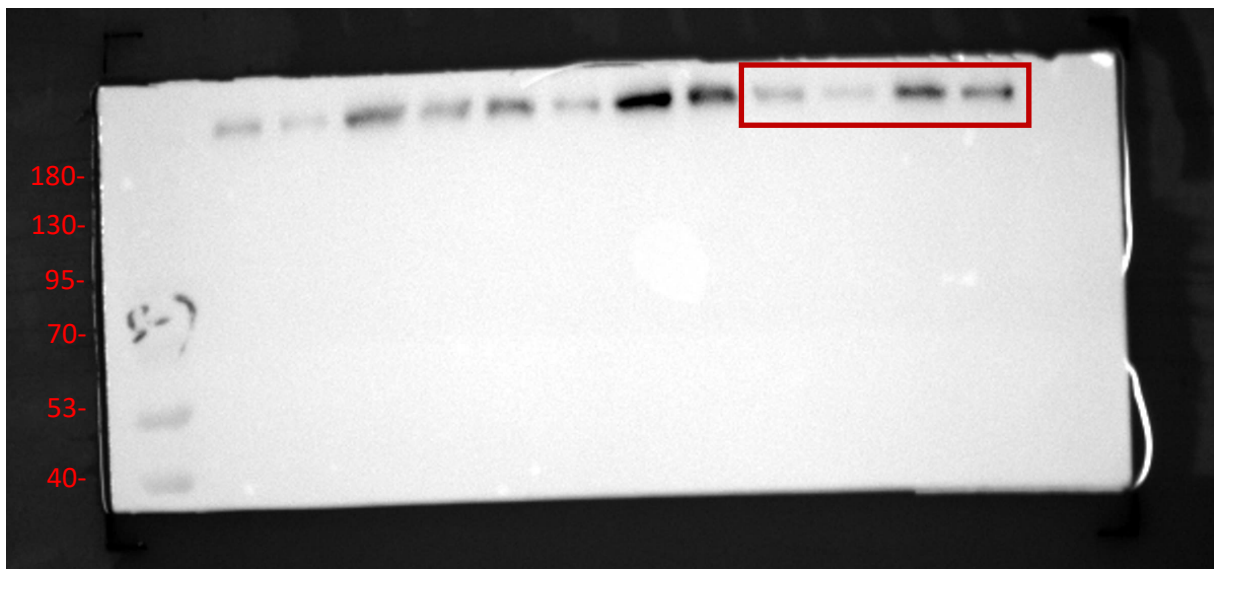


**Full unedited blot for Figure 4A:　GAPDH 37kDa**

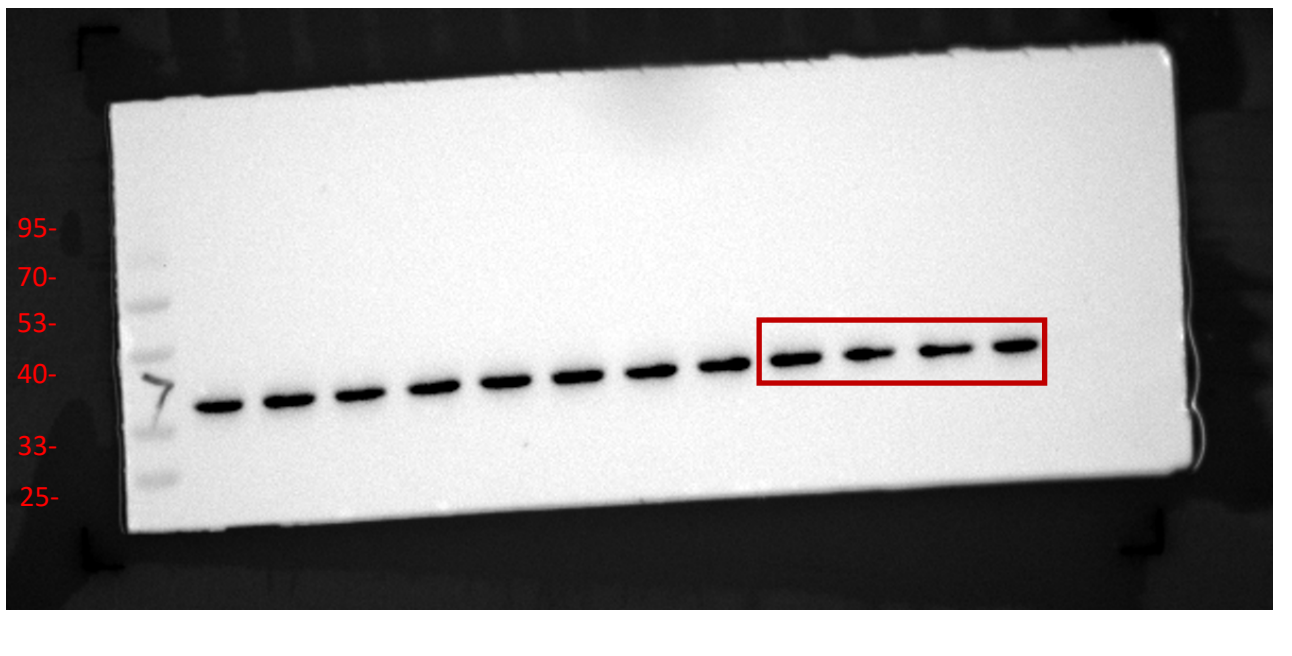

## Original Blot Figure 5

## Unedited Immunoblots

**Full unedited blot for Figure 4F：FUNDC1 17kDa**

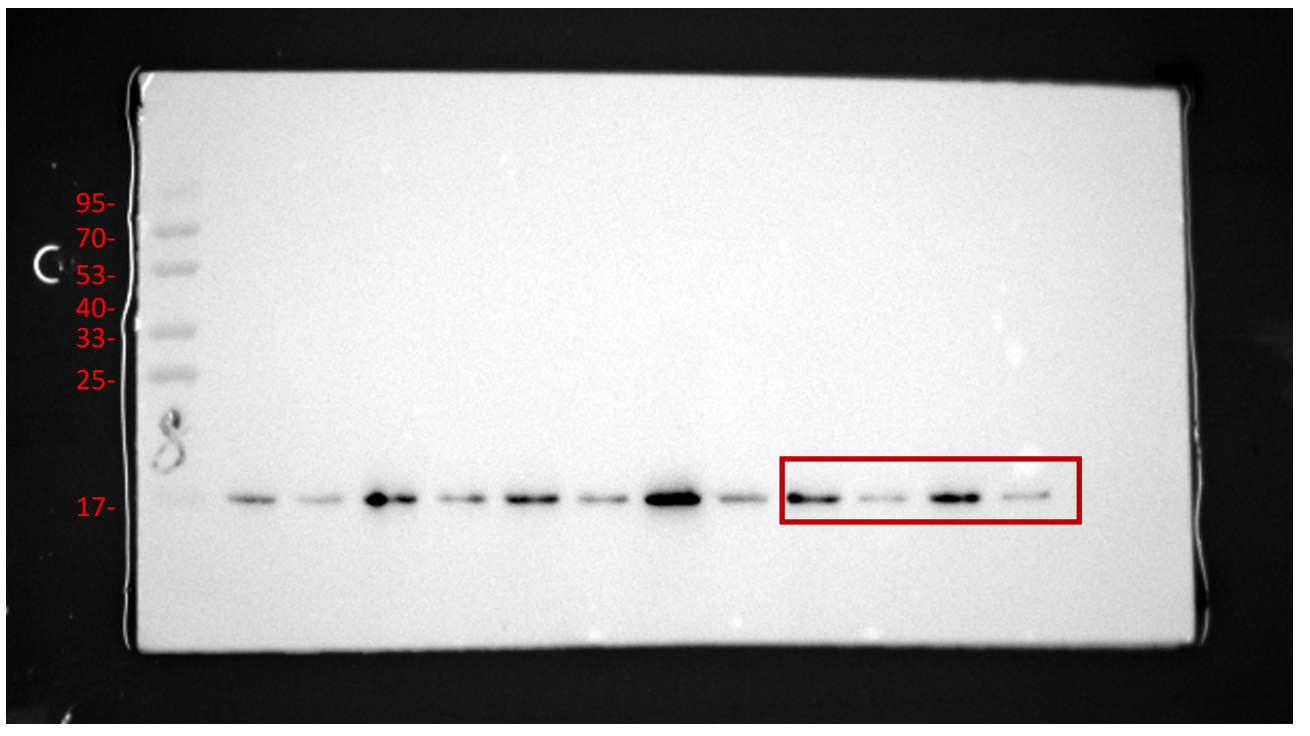


**Full unedited blot for Figure 4F：IP3R2 308kDa**

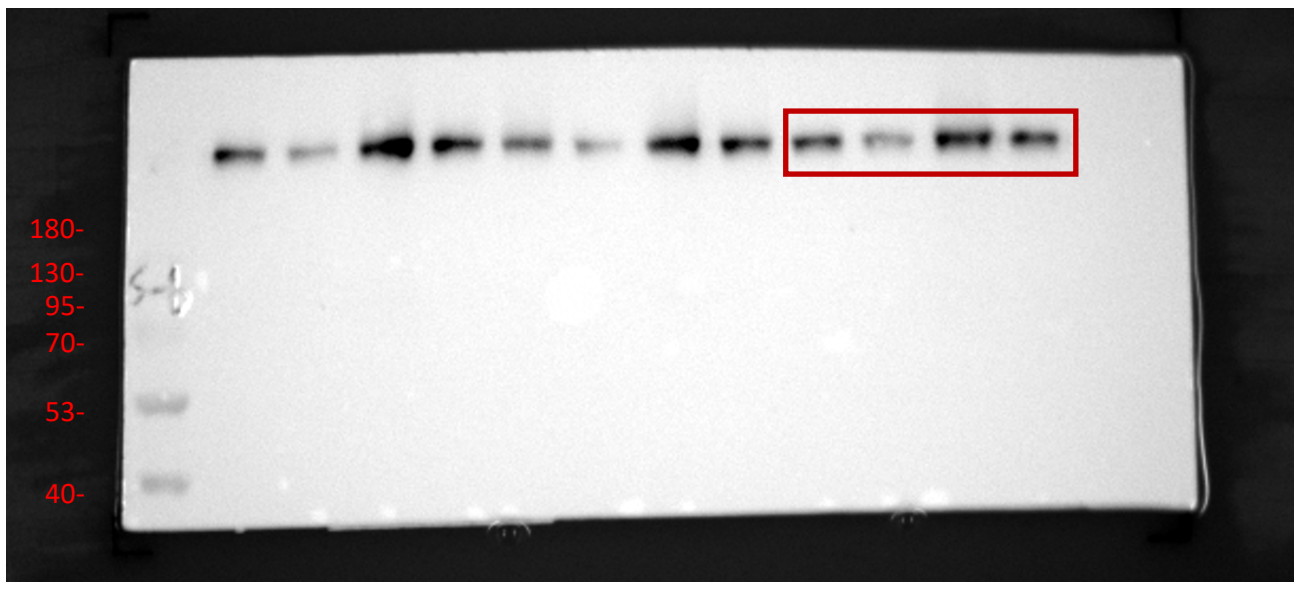


**Full unedited blot for Figure 4F：GAPDH 37kDa**

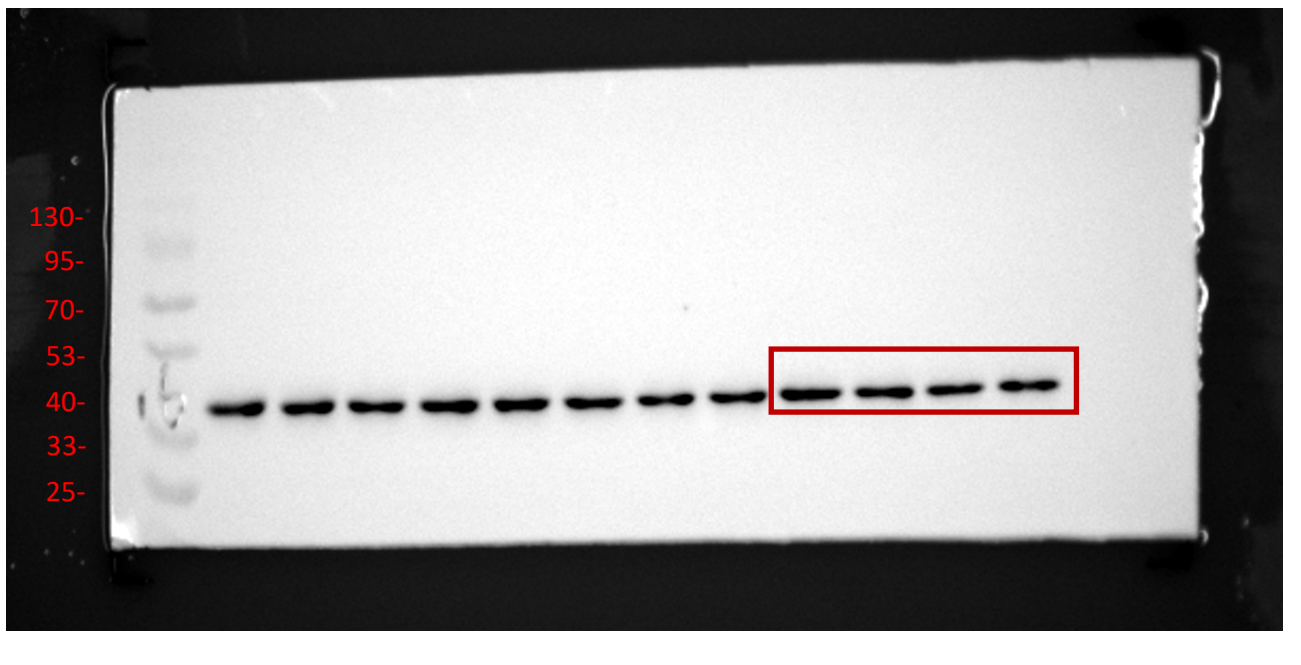

## Original Blot Figure 6

## Unedited Immunoblots

**Full unedited blot for Figure 4G: FUNDC1 17kDa**

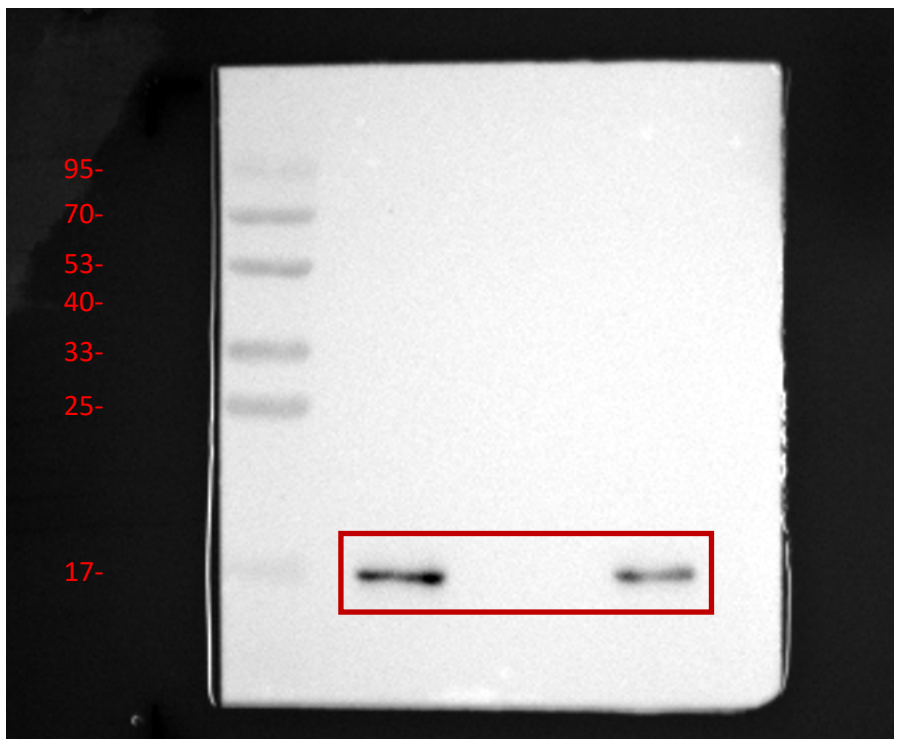


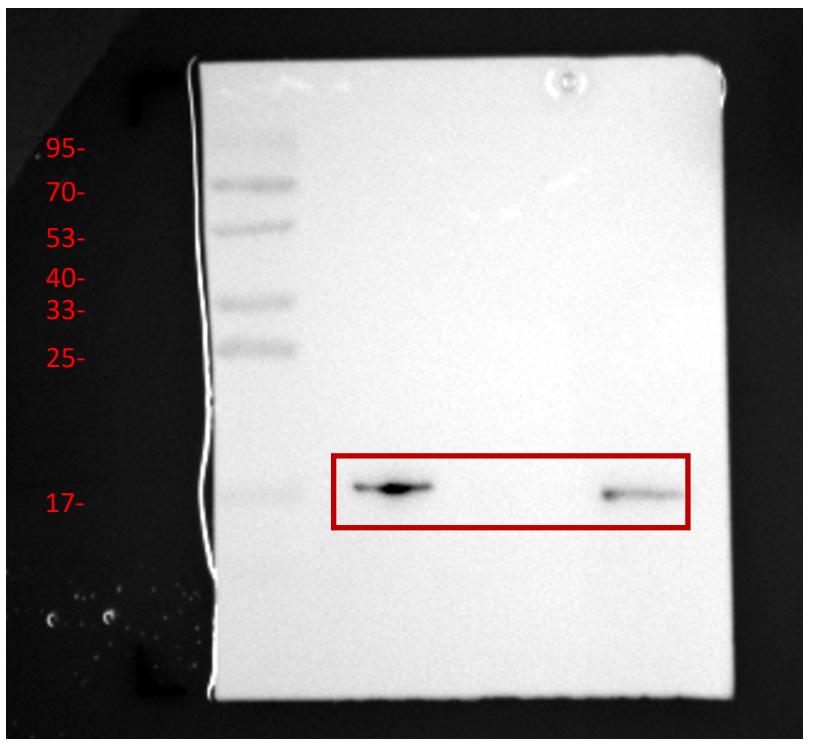


**Full unedited blot for Figure 4G: IP3R2 308kDa**

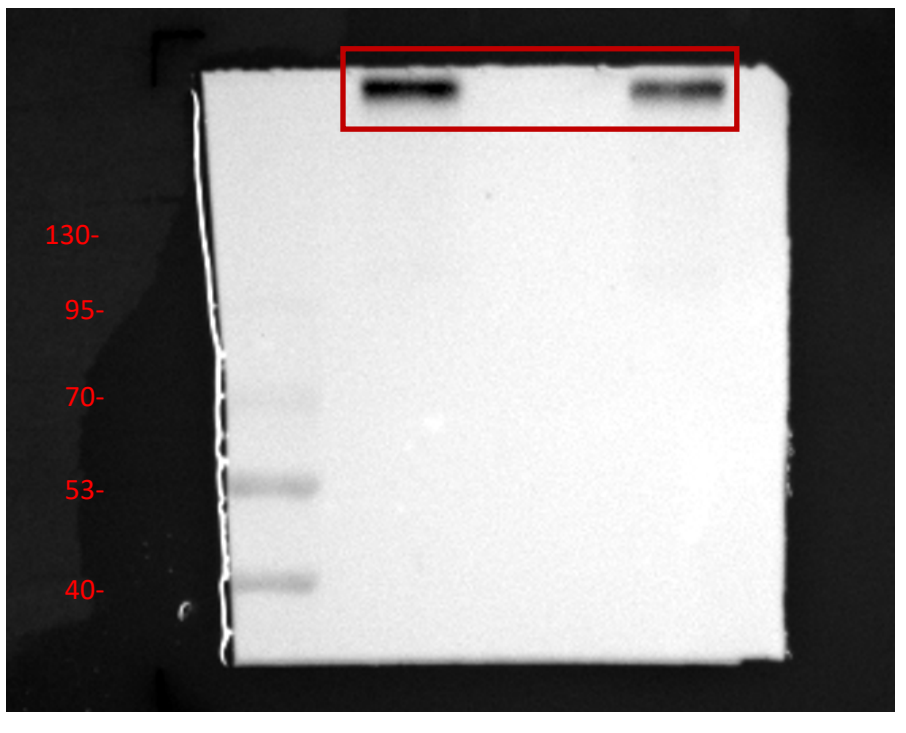


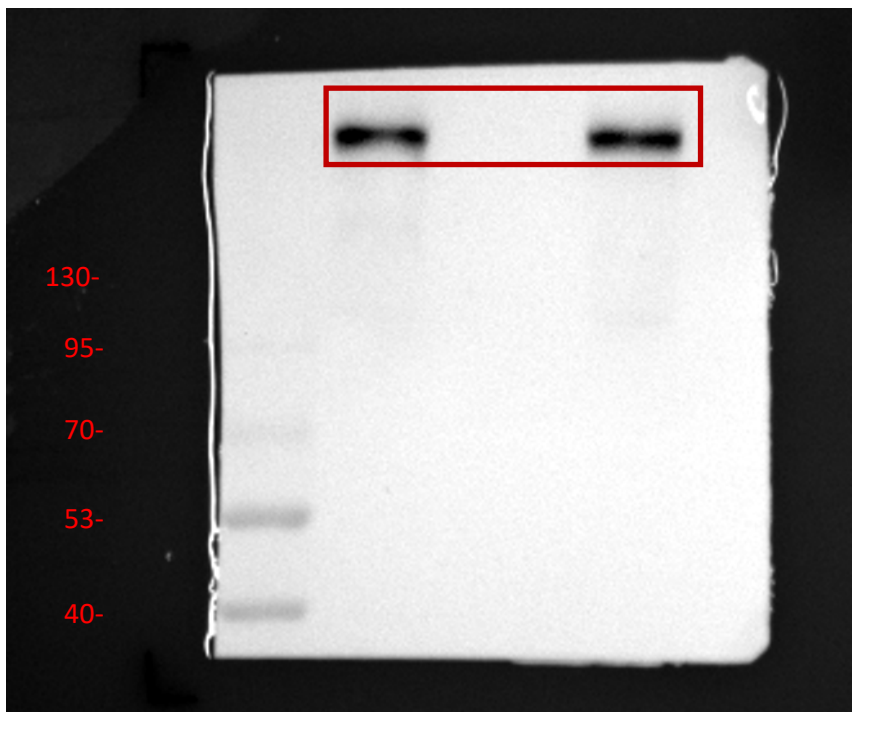

## Original Blot Figure 7

### Unedited Immunoblots

**Full unedited blot for Figure 5A: ANP 17kDa**

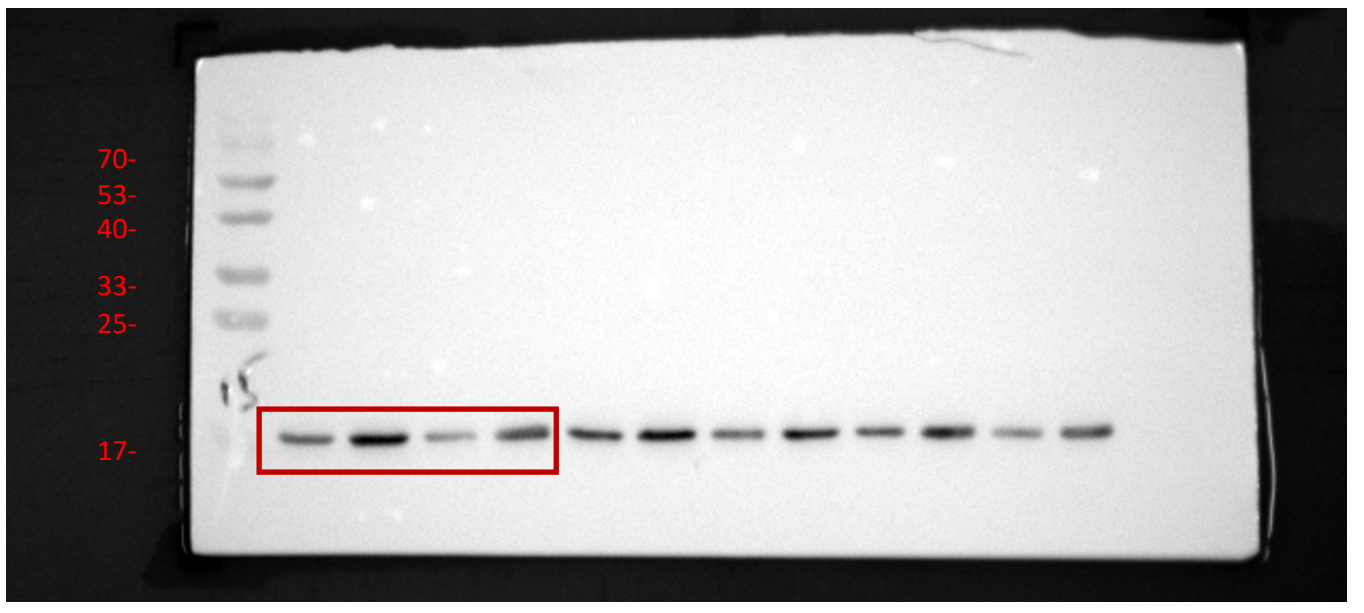


**Full unedited blot for Figure 5A: BNP 15kDa**

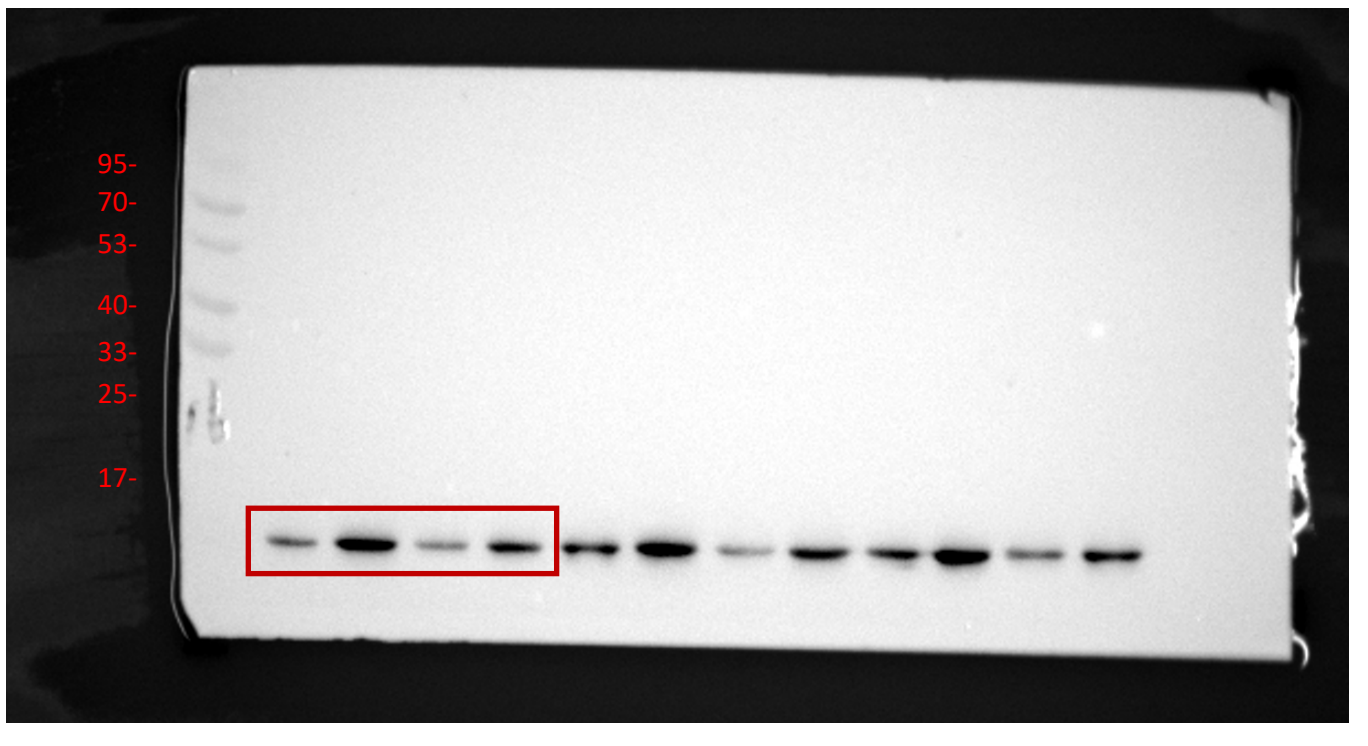


**Full unedited blot for Figure 5A: β-MHC 220kDa**

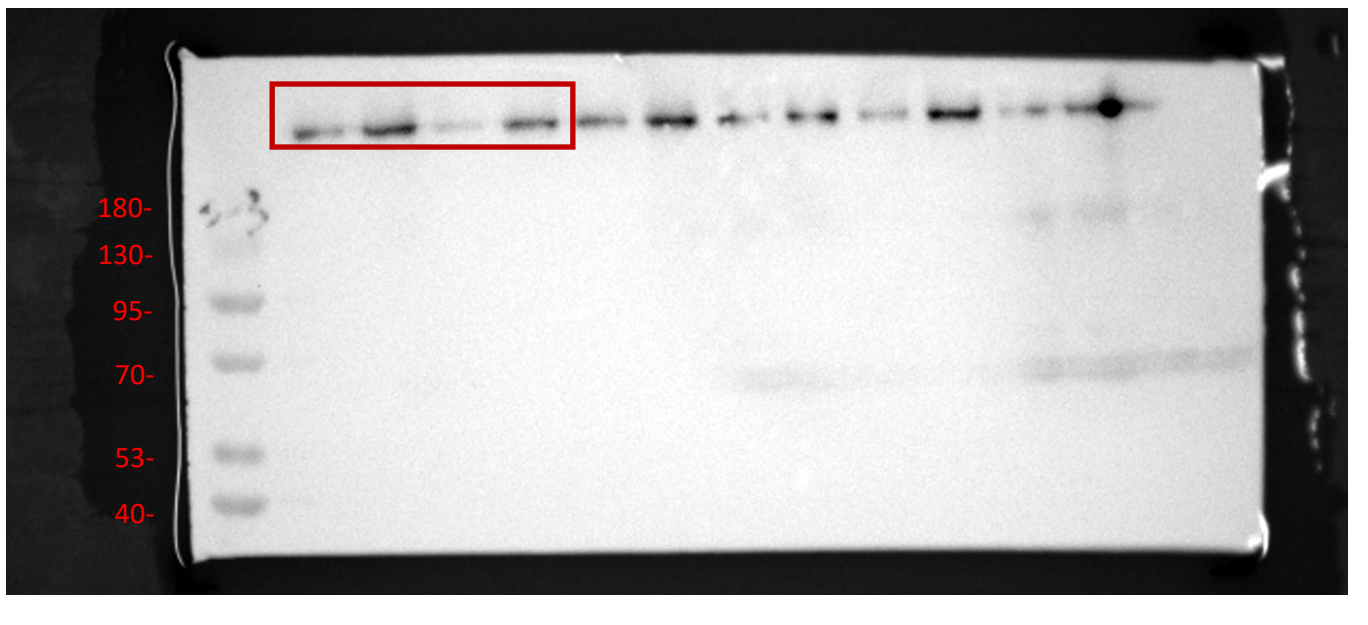


**Full unedited blot for Figure 5A: GAPDH 37kDa**

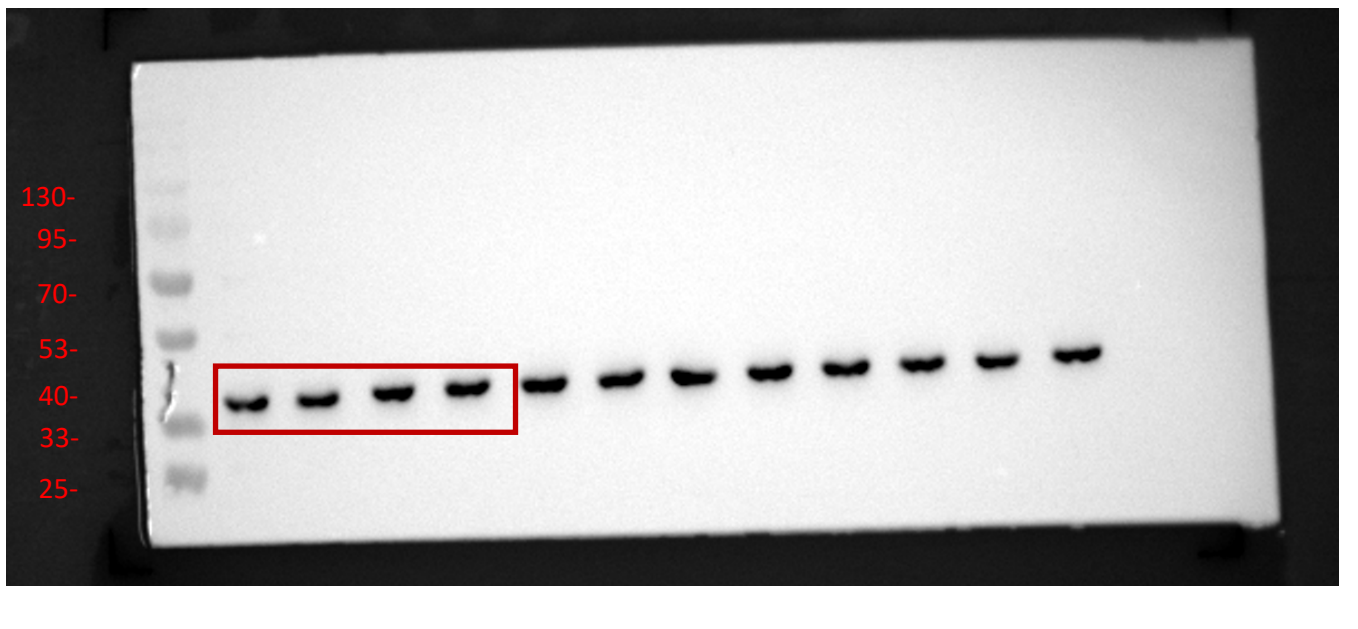

## Original Blot Figure 8

### Unedited Immunoblots

**Full unedited blot for Figure 5F：FUNDC1 17kDa**

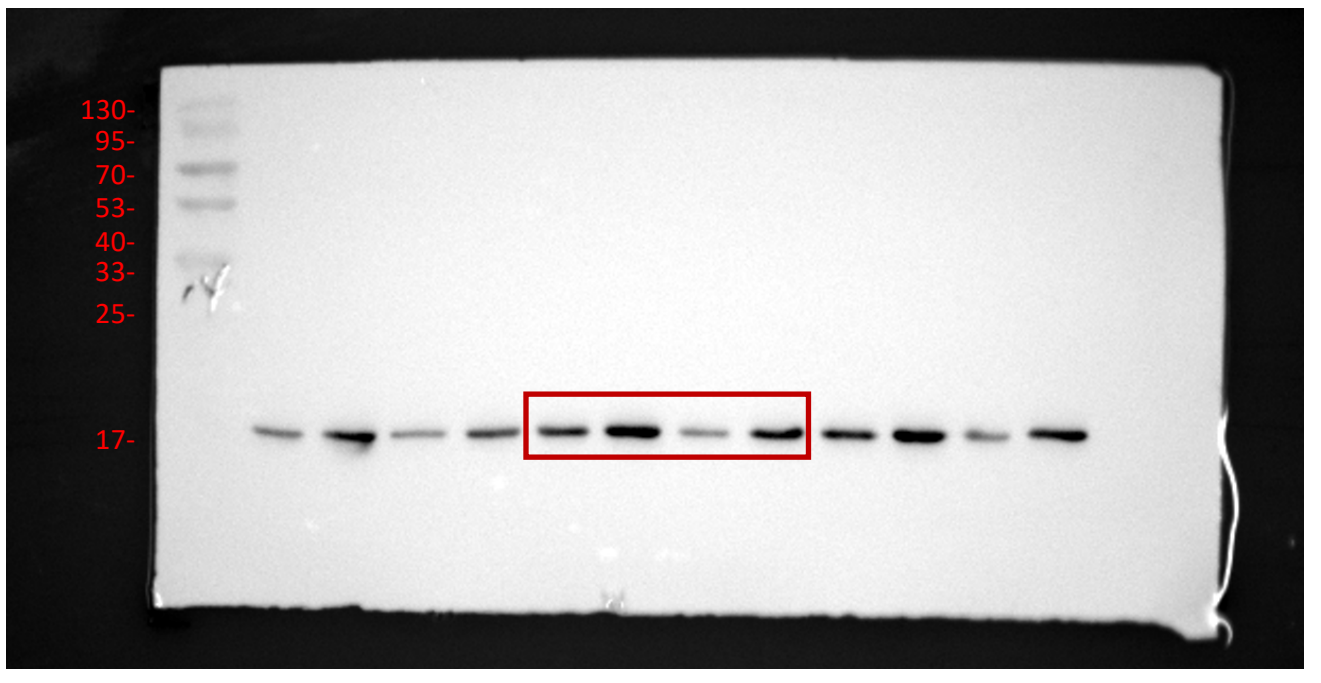


**Full unedited blot for Figure 5F： IP3R2 308kDa**

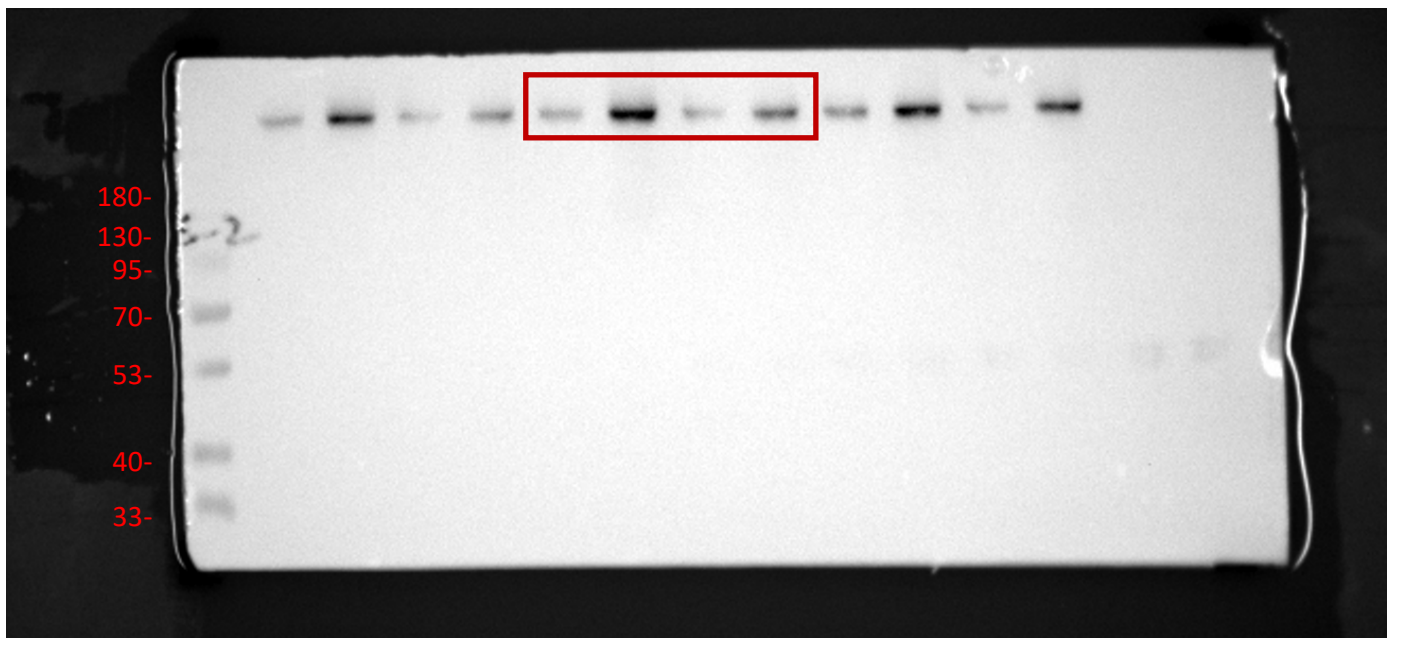


**Full unedited blot for Figure 5F： GAPDH 37kDa**

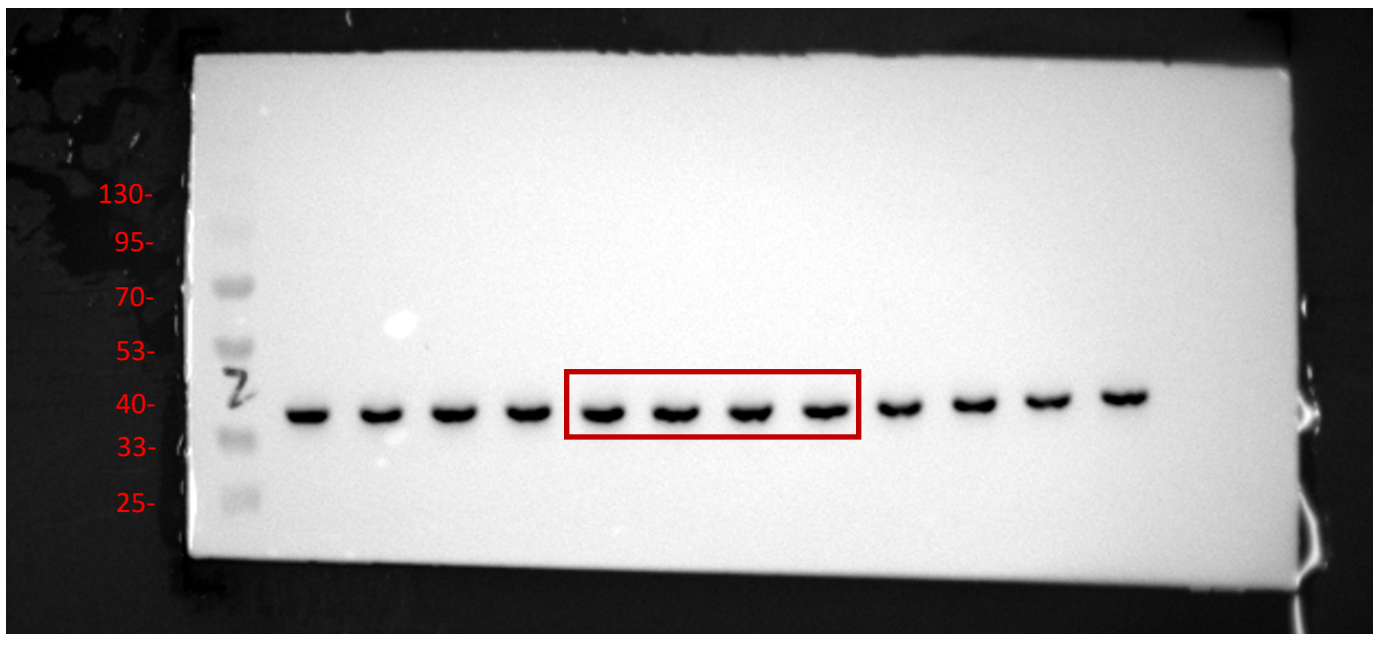

## Original Blot Figure 9

## Unedited Immunoblots

**Full unedited blot for Figure S6A：FUNDC1 17kDa**

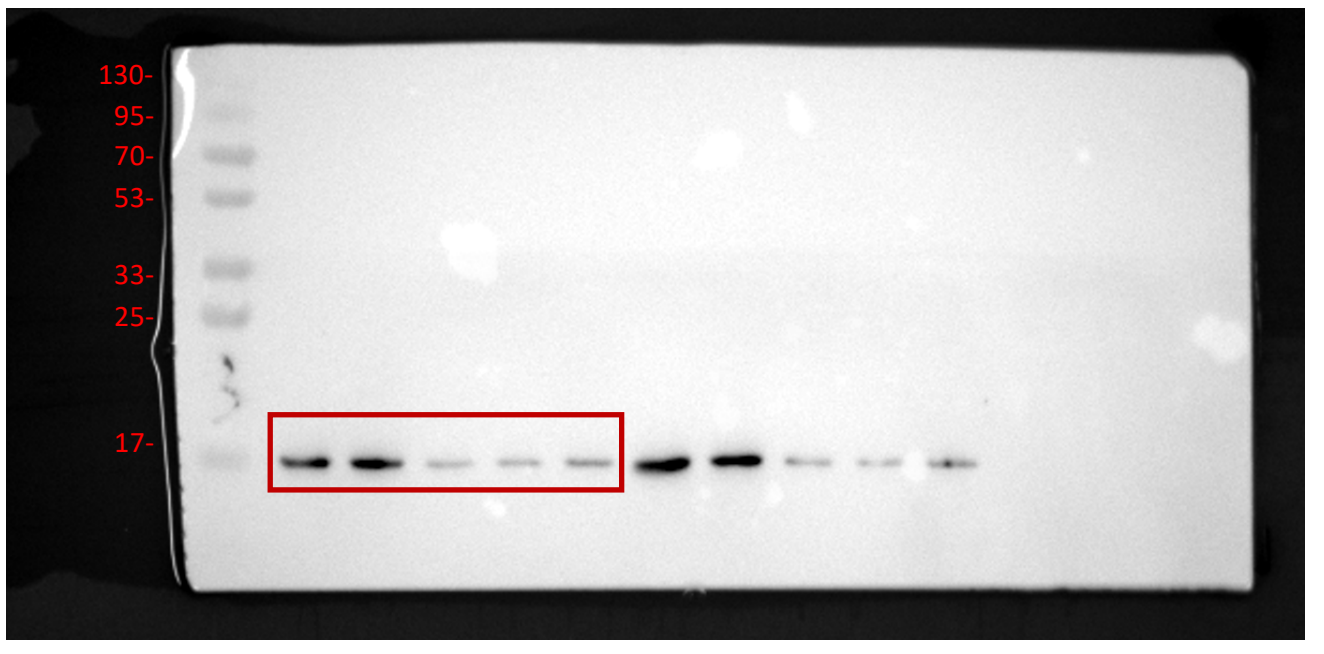


**Full unedited blot for Figure S6A：GAPDH 37kDa**

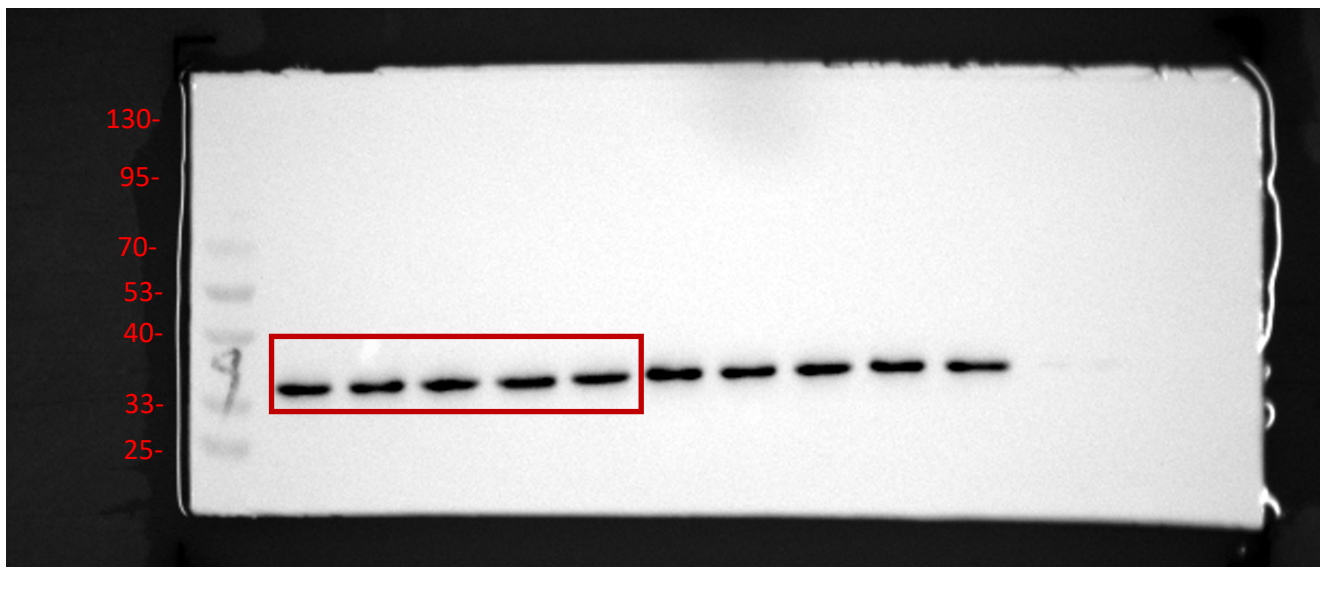

# Original Blot Figure 10

## Unedited Immunoblots

**Full unedited blot for Figure S6B：FUNDC1 17kDa**

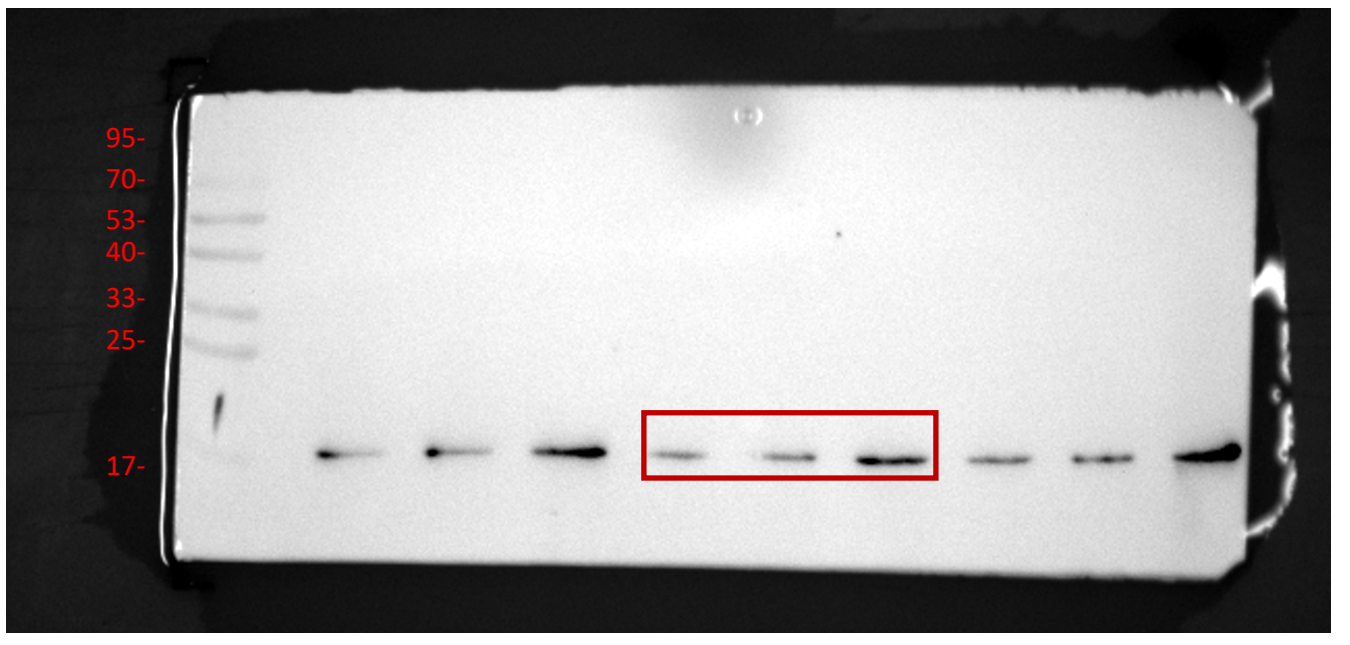


**Full unedited blot for Figure S6B： GAPDH 37kDa**

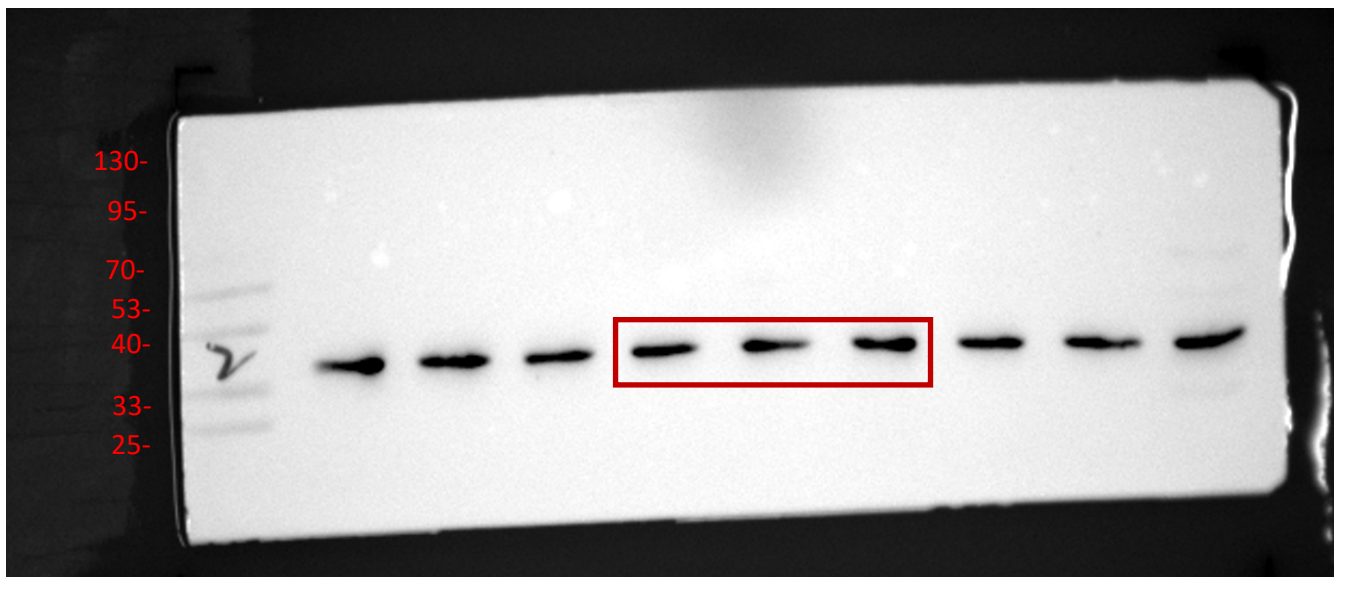

Supplement: Supplementary file 1 [file 41598_2026_56197_MOESM3_ESM.pdf]
