# Supplementary material for: Huoxue Qianyang Qutan Recipe limits cardiac remodeling by regulating FUNDC1/IP3R2 signaling pathway in obese hypertensive rats
Source: Sci Rep. 2026 Jun 4;16:25449. doi: 10.1038/s41598-026-56197-y (PMC13473496; doi:10.1038/s41598-026-56197-y)
Supplement: Supplementary file 2 [file 41598_2026_56197_MOESM2_ESM.docx]

**Supplemental methods**

**1. LC-MS/MS analysis**

**1.1 Chemicals and Reagents**

Methanol, acetonitrile, formic acid, and isopropyl alcohol were purchased from ANPEL (Shanghai, China). All organic solvents were of LC-MS grade. Ultra-pure water was prepared in-house using a Milli-Q water purification system (Millipore, Bedford, MA, USA).

**1.2 Sample Preparation and Extraction**

Samples were slowly thawed at 4 °C, and an appropriate volume (0.5–1.0 mL) was transferred into a centrifuge tube. Extraction solution (methanol-acetonitrile, 1:1, v/v) was added at twice the sample volume, followed by homogenization for 60 s and low-temperature ultrasonic extraction for 30 min. The mixture was centrifuged at 12,000 rpm for 10 min at 4 °C, then allowed to stand at −20 °C for 1 h for protein precipitation. After a second centrifugation at 12,000 rpm for 10 min at 4 °C, the supernatant was dried under vacuum. The residue was reconstituted with 0.1 mL of 50% acetonitrile aqueous solution, homogenized, and centrifuged at 12,000 rpm for 10 min at 4 °C. The final supernatant was collected for instrumental analysis.

**1.3 LC-MS/MS Analysis**

The extracted samples were analyzed on an UPLC-Orbitrap-MS system consisting of a Vanquish UPLC system and a Q Exactive HFX mass spectrometer (Thermo Fisher Scientific, Waltham, MA, USA).

UPLC conditions

Analytical column: Waters HSS T3 column (100 × 2.1 mm, 1.8 μm); column temperature: 40 °C; flow rate: 0.3 mL/min; injection volume: 2 μL; mobile phase: A (Milli-Q water containing 0.1% formic acid), B (acetonitrile containing 0.1% formic acid). Gradient elution program: 0–1 min, 100% A; 1–12 min, linear gradient to 5% A and 95% B; 12–13 min, hold at 5% A and 95% B; 13.1 min, return to 100% A; 13.1–17 min, hold at 100% A for re-equilibration.

**1.4 MS conditions**

High-resolution mass spectrometry (HRMS) data were acquired in Full MS/ddMS² mode with a heated electrospray ionization (HESI) source. The ESI source operating parameters were set as follows: sheath gas flow rate: 40 arb; auxiliary gas flow rate: 10 arb; spray voltage: +3000 V/−2800 V; capillary temperature: 350 °C; ion transfer tube temperature: 320 °C. Full scan m/z range: 70–1050 Da; MS1 resolution: 70,000; MS2 resolution: 17,500.

**2. Network pharmacology analysis**

**2.1 Potential targets of main compounds in HQQR and targets associated with OBH**

Potential targets of main compounds in HQQR were collected from TCM systematic pharmacology analysis platform (TCMSP, http://lsp.nwu.edu.cn/tcmsp.php)^1^, STITCH database (STITCH, https://stitch.embl.de/)^2^, PubChem chemistry database (PubChem, https://pubchem.ncbi.nlm.nih.gov/)^3^, and the universal protein resource (UniProt, https://www.uniprot.org/) ^4^.

Targets associated with OBH were collected by searching the human gene database (GeneCards, https://www.genecards.org/)^5^, DisGeNET database (DisGeNET, http://www.disgenet.org/)^6^, and Uniprot. The keywords were “obesity-associated hypertension”, “obesity-related hypertension”, and “obesity AND hypertension”.

**2.2 Construction of protein-protein interaction (PPI) network**

Core targets of herbs in HQQR and OBH-related genes were imported into the STRING database (STRING, https://string-db.org/)^7^ for PPI network construction and data analysis. The organism was set to “Homo Sapiens”, and the minimum required interaction score is medium confidence. Input PPI data into Cytoscape3.7.2^8^ for visualization, and construct PPI network. The core targets of active ingredients of HQQR were screened using Merge function, and the key active ingredients-core targets network were drawn to analyze the potential mechanisms of HQQR in the treatment of OBH.

**2.3 KEGG pathway and GO enrichment analysis**

To investigate the key signaling pathways and biological processes of HQQR in the treatment of OBH, Metascape database (Metascape, http://metascape.org/)^9^ was used to perform KEGG signaling pathway as well as GO enrichment analysis. Dot plot was plotted by https://www.bioinformatics.com.cn, an online platform for data analysis and visualization^10^. The horizontal axis represents the gene ratio, while the vertical axis represents the enriched pathway name. The color scale indicates different thresholds of the p-value, and the size of the dot indicates the number of genes corresponding to each pathway.

**References**

1	Ru, J. *et al.* TCMSP: a database of systems pharmacology for drug discovery from herbal medicines. *J Cheminform* **6**, 13 (2014). <https://doi.org/10.1186/1758-2946-6-13>

2	Szklarczyk, D. *et al.* STITCH 5: augmenting protein-chemical interaction networks with tissue and affinity data. *Nucleic Acids Res* **44**, D380-384 (2016). <https://doi.org/10.1093/nar/gkv1277>

3	Kim, S. *et al.* PubChem in 2021: new data content and improved web interfaces. *Nucleic Acids Res* **49**, D1388-d1395 (2021). <https://doi.org/10.1093/nar/gkaa971>

4	UniProt: a worldwide hub of protein knowledge. *Nucleic Acids Res* **47**, D506-d515 (2019). <https://doi.org/10.1093/nar/gky1049>

5	Stelzer, G. *et al.* The GeneCards Suite: From Gene Data Mining to Disease Genome Sequence Analyses. *Curr Protoc Bioinformatics* **54**, 1.30.31-31.30.33 (2016). <https://doi.org/10.1002/cpbi.5>

6	Piñero, J. *et al.* DisGeNET: a comprehensive platform integrating information on human disease-associated genes and variants. *Nucleic Acids Res* **45**, D833-d839 (2017). <https://doi.org/10.1093/nar/gkw943>

7	Szklarczyk, D. *et al.* STRING v11: protein-protein association networks with increased coverage, supporting functional discovery in genome-wide experimental datasets. *Nucleic Acids Res* **47**, D607-d613 (2019). <https://doi.org/10.1093/nar/gky1131>

8	Shannon, P. *et al.* Cytoscape: a software environment for integrated models of biomolecular interaction networks. *Genome Res* **13**, 2498-2504 (2003). <https://doi.org/10.1101/gr.1239303>

9	Zhou, Y. *et al.* Metascape provides a biologist-oriented resource for the analysis of systems-level datasets. *Nature communications* **10**, 1523 (2019). <https://doi.org/10.1038/s41467-019-09234-6>

10	Tang, D. *et al.* SRplot: A free online platform for data visualization and graphing. *PLoS One* **18**, e0294236 (2023). <https://doi.org/10.1371/journal.pone.0294236>

**Figure S1. LC–MS/MS profiling of HQQR**

(A–B) Base peak chromatograms (BPC) of HQQR acquired in positive (A) and negative (B) ion modes. The BPC displays the intensity of the most abundant ion at each retention time, providing a simplified and noise-reduced overview of the major chemical constituents present in HQQR.

**Figure S2. MS/MS Spectrums of key identified compounds in HQQR by LC‑MS/MS** **in positive ion modes (Set 1)**

(A) Hirsutine; (B) Rhynchophylline; (C) Dihydrotanshinone I; (D) Tanshinone IIA; (E) Senkyunolide A; (F) Betaine; (G) Chlorogenic acid; (H) Isoquercitrin; (I) Luteolin; (J) Ferulic acid.

**Figure S3.** **MS/MS Spectrums of key identified compounds in HQQR by LC‑MS/MS in negative modes (Set 2)**

(A) Danshensu; (B) Salvianolic acid A; (C) Quercetin‑3‑gentiobioside; (D) Cryptochlorogenic acid; (E) Protocatechuic acid; (F) Lithospermic acid; (G) Salvianolic acid C; (H) p‑Coumaric acid; (I) Gallic acid; (J) Rosmarinic acid.

**Figure S4. Network pharmacology analysis of HQQR in the treatment of obese hypertension (OBH)**

(A) Classification of chemical constituents in HQQR identified by LC–MS/MS based on ClassyFire chemical taxonomy. (B) Venn diagram showing the overlap between potential targets of HQQR and OBH targets. (C) Protein–protein interaction (PPI) network of 16 core targets identified by topological (centrality) analysis. (D) Compound–target network illustrating the interactions between major active components of HQQR and their corresponding targets. (E) Kyoto Encyclopedia of Genes and Genomes (KEGG) pathway enrichment analysis of the core targets. (F–G) Gene Ontology (GO) enrichment analysis of the core targets, including biological process (BP, green), cellular component (CC, orange), and molecular function (MF, blue).

**Figure S5. Establishment the concentration of angiotensin II**

Cell viability assay evaluating the effects of different angiotensin II concentrations on cardiomyocytes (n = 5).

**Figure S6. Establishment of FUNDC1 knockdown and overexpression cell models**

(A) Western blot analysis of FUNDC1 protein expression in cardiomyocytes transfected with shNC or three independent FUNDC1 shRNA constructs (shFUNDC1-1, shFUNDC1-2, shFUNDC1-3), and corresponding quantification of FUNDC1 protein levels normalized to GAPDH (n = 3). Original blots are presented in Original Blot Figure 9. (B) Western blot analysis of FUNDC1 protein expression in cardiomyocytes transfected with empty vector or FUNDC1 overexpression plasmid (oeFUNDC1), and corresponding quantification of FUNDC1 protein levels normalized to GAPDH (n = 3). Original blots are presented in Original Blot Figure 10. Data are presented as mean ± SD. ** and *** respectively means *P* < 0.01, and *P* < 0.001.
